# The contribution of the meningeal immune interface to neuroinflammation in traumatic brain injury

**DOI:** 10.1186/s12974-024-03122-7

**Published:** 2024-05-27

**Authors:** Alaa Y. Mokbel, Mark P. Burns, Bevan S. Main

**Affiliations:** Department of Neuroscience, Georgetown University Medical Center, New Research Building-EG11, 3970 Reservoir Rd, NW, Washington, DC 20057 USA

**Keywords:** Meninges, Innate immunity, Adaptive immunity, Neuroinflammation, TBI

## Abstract

Traumatic brain injury (TBI) is a major cause of disability and mortality worldwide, particularly among the elderly, yet our mechanistic understanding of what renders the post-traumatic brain vulnerable to poor outcomes, and susceptible to neurological disease, is incomplete. It is well established that dysregulated and sustained immune responses elicit negative consequences after TBI; however, our understanding of the neuroimmune interface that facilitates crosstalk between central and peripheral immune reservoirs is in its infancy. The meninges serve as the interface between the brain and the immune system, facilitating important bi-directional roles in both healthy and disease settings. It has been previously shown that disruption of this system exacerbates neuroinflammation in age-related neurodegenerative disorders such as Alzheimer’s disease; however, we have an incomplete understanding of how the meningeal compartment influences immune responses after TBI. In this manuscript, we will offer a detailed overview of the holistic nature of neuroinflammatory responses in TBI, including hallmark features observed across clinical and animal models. We will highlight the structure and function of the meningeal lymphatic system, including its role in immuno-surveillance and immune responses within the meninges and the brain. We will provide a comprehensive update on our current knowledge of meningeal-derived responses across the spectrum of TBI, and identify new avenues for neuroimmune modulation within the neurotrauma field.

## Background

Traumatic brain injury (TBI) is a leading cause of death and disability, posing a significant socioeconomic and public health burden, with an estimated 64–74 million people sustaining a TBI each year [1, 2]. While the prevalence of TBI is centered around young adults and the elderly [3, 4], the consequences of TBI are more severe in aged populations. Slower recovery, worse functional, cognitive, and psychosocial outcomes, all highlight the influence of age on overall TBI pathogenesis [5–11]. Strikingly, there were 69,473 TBI-related deaths in the United States alone in 2021 [12], emphasizing the need for disease modifying interventions. There are no comprehensive pharmacological treatments for TBI, with the diversity of injury still considered a significant barrier toward the translation of effective therapeutics [13]. Although TBI can be influenced by many variables, it is accepted that neuroinflammation contributes to negative outcomes after TBI. Evidence suggests that the synergistic functioning of innate and adaptive immune cells, crucial for orchestrating and sustaining a healthy brain microenvironment, becomes dysfunctional after TBI. This dysfunction results in sustained, uncontrolled neuroinflammatory responses which detrimentally affect outcomes and recovery timeframes. Indeed, therapies targeting inflammatory responses display efficacy in preclinical and single-center trials; however, they fail to show improvement in larger multicenter clinical trials [14, 15]. This may be due to the fact that the majority of preclinical TBI studies tend to analyze these cellular responses individually, with interventions focused on targeting a singular cell modality. It is now evident that neuroinflammatory responses in TBI are not confined to a single cell type, and it is more likely that a combination of complex cellular interactions determines the nature of inflammatory cascades. Therefore, in order to develop more effective therapeutics, we require a greater understanding of the bi-directional crosstalk between the innate and adaptive immune response and how they contribute holistically to TBI-induced neuroinflammation.

## Neuroinflammation in TBI

The complexity and challenges in understanding cellular interactions and signaling cascades in TBI is largely due to the heterogenous nature of injury. Primary injury causes brain lesions from fractures, intracranial hemorrhage, epidural and subdural hematoma, brain contusion and direct mechanical damage to neural tissue. Secondary injury occurs immediately following head impact, initiating cascades including neuroinflammation that can persist for weeks and even years, contributing to neurological impairment. Temporally, primary injury results in blood–brain barrier (BBB) dysfunction, neural damage, and release of endogenous damage-associated molecular patterns (DAMPs). These DAMPs subsequently engage pattern recognition receptors (PRR), such as the toll like receptors (TLRs) on innate (microglia/astrocytes) or adaptive (myeloid/lymphoid) cells, leading to their immune activation. The molecular diversity of DAMPs binding PRRs is vast (reviewed in [16]), and their significance after trauma is critical, with DAMP level correlating with injury severity and inversely related to clinical outcomes [17]. Indeed, clinical insights offer invaluable translational knowledge to identify secondary injury triggers and processes, information that may identify avenues for therapeutic intervention.

### Clinical hallmarks of neuroinflammation in TBI

Physical trauma to the brain causes BBB disruption, with increased fibrinogen, immunoglobulin and heightened cerebrospinal fluid (CSF) to serum albumin quotients detectable within hours of clinical evaluation, persisting for weeks or even years [18–26]. Increases in serum and CSF levels of brain specific glial fibrillary acid protein (GFAP), ubiquitin carboxyl-terminal hydrolase isozyme L1 (UCH-L1) and S100 calcium-binding protein B (S100B) are correlated with early barrier permeability [20, 27]. Similarly, increased CSF levels of matrix metalloproteinases (MMPs) [28] and the complement mediators C3, factor B and sC5b-9 are also associated with BBB dysfunction [23, 29]. The CSF of severe TBI patients also contains DAMPs, PRRs and mediators downstream from PRR activation, with elevated levels of high mobility group protein B1 (HMGB1) [30–32], double stranded DNA (dsDNA), absent in melanoma 2 (AIM2), apoptosis-associated speck-like protein (ASC), NLR Family Pyrin Domain Containing 1 (NLRP1), and caspase-1 [33, 34]. Fluid biomarkers (CSF/serum/blood) show temporal increases in cytokines and chemokines, including members of respective interferon, interleukin, tumor necrosis factor, transforming growth factor and C-C motif ligand families (Table 1).

**Table 1:** Cytokines and chemokines modulating neuroinflammation in clinical TBI

Immune signature/trigger	Time
Fluid biomarkers	
IFNγ	↑ 1–5 days after injury [35, 36], ↑ up to 12 months [37]
TNF	↑ 6 h–2 weeks days [24, 35, 38–50], ↑ up to 12 months [37]
IL-1β	↑ 1 day–1 month [39, 46, 51–59]
IL-6	↑ 6 h–2 weeks [24, 36, 46, 47, 49–52, 56, 58–68], ↑ up to 6 months [69]
IL-10	↑ 6 h–3 days [24, 39, 46, 51, 68, 70–73]
IL-8 (CXCL8)	↑ 2 h–5 days [48, 50, 51, 59, 69, 74], ↑ up to 12 months [37]
IL-12p70	↑ 1–3 days, peaks at days 3–5 [46, 50, 51]
TGFβ	↑ 1–21 days after trauma [75, 76]
CCL2	↑ 1–10 days [46, 77, 78], ↑ up to 3 months [58]
CCL3	↑ 1–3 days [46, 51]
CXCL8 (IL-8)	↑ 1–4.5 days [46, 51, 63, 79]
IL-18	↑ up to 10 days [80]
sIL-2R	↑1–21 days [81]
IL-17, IL-22	↑ day 5 after injury [35], ↑ up to 12 months [37]
IL-2	↑ 24 h post injury [67]
IL-9	↑ admission–12 months [37]
IL-4	↑ within 48 h post injury [59]
iNOS	↑24 h post injury [47]
NADPH	↑24 h post injury [47]
COX-2	↑24 h post injury [47]
Tissue	
IFNγ	↑ 17 min–5 days injury [70]
TNF	↑ 17 min–5 days injury [70]
IL-1β	↑ 6 h–5days after injury [70]
IL-6	↑ 17 min after injury [56, 70]
CCL2	↑ 3 h–15 days post-injury [78]
CCL3	↑ 3 h–15 days post-injury [78]
IL-8 (CXCL8)	↑ 3 h–15 days post-injury [70, 78]
IL-2	↑ 17 min–5 days injury [70]
NOX2	↑12–24 h post injury [82]
NOX4	↑12–48 h post injury [82]

Indeed, tissue samples from patients with TBI indicate that this inflammatory response is composed of both innate and adaptive cellular elements, including monocytes/macrophages, reactive microglia, polymorphonuclear cells, and CD4^+^, CD8^+^ T cells [83–88]. Using positron emission tomography (PET) scans, TBI patients show elevated translocator protein (TSPO) expression in microglia, demonstrating microglial activation which could be seen up to 17 years after injury [89, 90]. Indeed, in brain tissue, activated microglia (CD68^+^, CD11b^+^, TMEM119^+^) are highly expressed after injury across acute and chronic timepoints [83, 85, 91–94], alongside markers for toll like receptor (TLR) signaling including TLR4 and myeloid differentiation primary response 88 (Myd88) [95, 96]. Peripheral cell contributions are observed in blood samples after TBI, with acute decreases of the number CD4^+^ and CD8^+^ and natural killer (NK) cells, followed by transient increases in T regulatory (Treg) cells after injury [35, 97–100]. The temporal series of these responses may be influenced by injury severity, with expansion of Th17-type CD4 T cells alongside IL-17 and IL-22, seen at 5 days post injury [35]. Concurrently NK cells have reduced T-bet expression and lower IFNγ and TNFα, all indicating cell specific responses after TBI [35].

### Hallmarks of neuroinflammation in animal models of TBI

**Fig. 1:**
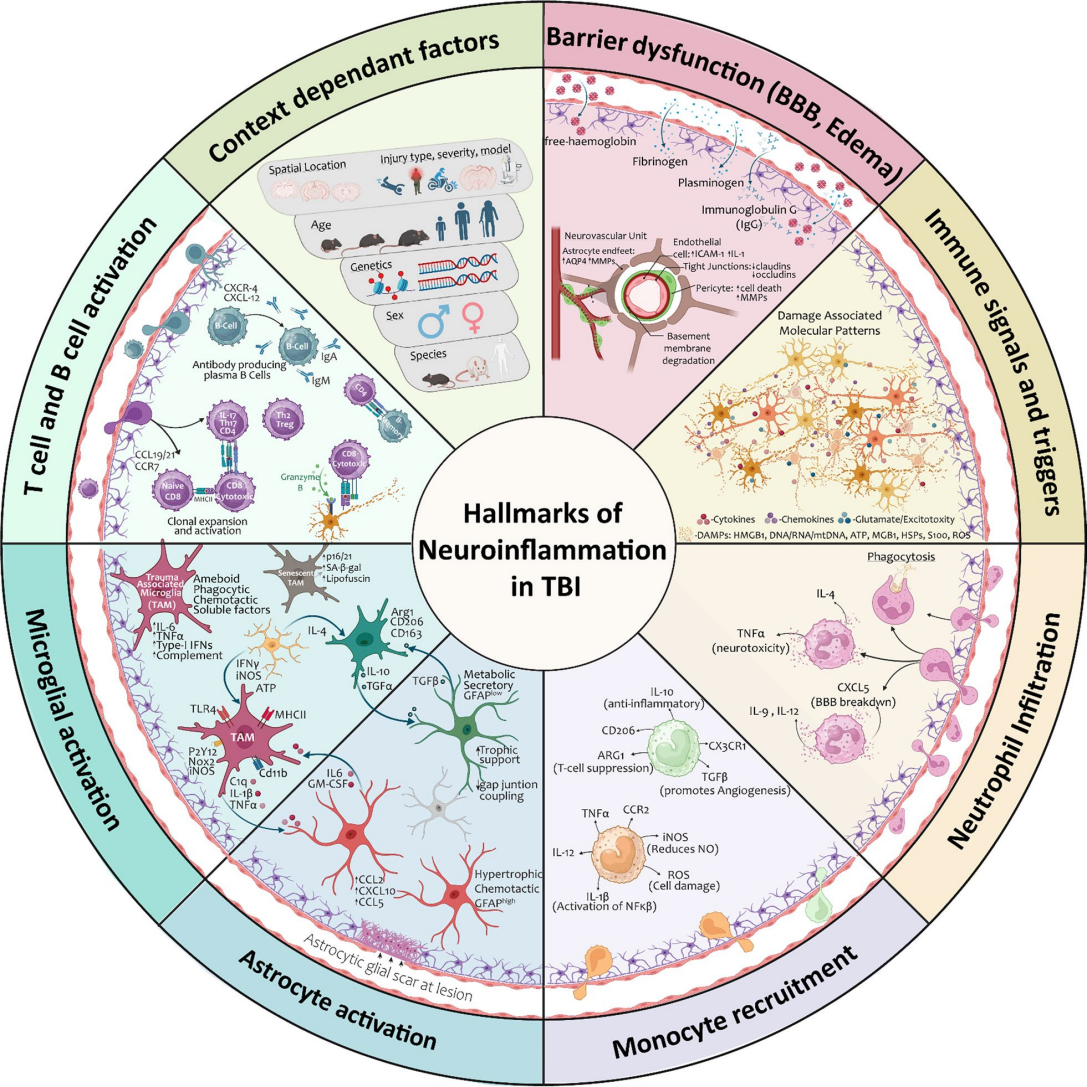
Hallmarks of neuroinflammation in Traumatic Brain Injury. Summary of hallmark characteristics that encompass the holistic nature of TBI-induced neuroinflammation. Based on decades of clinical research and mechanistic knowledge gained from the diverse range of injury models, key neuroinflammatory events include: barrier dysfunction, immune triggers and signaling, neural damage or release of soluble factors, neutrophil infiltration, monocyte recruitment, resident CNS microglial and astrocyte responses and activation of T and B cell responses. Importantly, all areas of this neuroinflammatory wheel can be influenced by context dependent factors, and should be considered across the spectrum of the neurotrauma field

Animal models have proven invaluable for studying TBI and unraveling the complicated mechanisms underlying both primary and secondary injuries. Similar to clinical studies, pre-clinical models show BBB permeability within hours, with spontaneous closure at approximately 7–10 days post-injury [101–105]. Mechanistically, TBI has been shown to affect processes at the neurovascular unit, with reductions in tight junction proteins (claudins, occludins) and pericyte loss [104, 106–108]. Across the spectrum of TBI models, various signals and triggers are elevated following injury, including DAMPS, cytokines, chemokines and soluble factors similar to those previously identified in clinical studies. Critically, their time and cell dependent function can be influenced by a myriad of context dependent factors (Fig. 1). Broadly, experimental TBI increases levels of DAMPs including HMGB1, ATP, heat-shock, GM-CSF, mtDNA and S100 proteins and their receptors such as TLRs and receptors of advanced glycosylation end-products (RAGE) and purinergic receptors. In addition, alterations in levels of proinflammatory cytokines TNF-α, IL-1β, IL-6, IL-18, IFNγ, Type-I interferons (IFNs), anti-inflammatory cytokines Arg-1, IL-4, IL-10, TGF-β and chemokines IL-8, MCP1, CCL2, CCL5, CXCL2 are all involved within the holistic nature of inflammation in TBI models (detailed review in [14, 109, 110]).

### Innate CNS responses

Microglia are resident central nervous system (CNS) immune cells, making about 10–13% of the cell population in the mouse and human brain [111, 112]. Their primary functions can be grouped to include surveillance, phagocytosis, and the secretion of soluble factors [113]. To determine functionality, microglia react to changes and context specific stimuli within CNS, eliciting a diverse array of tailored responses. Indeed, advancements in technologies (transcriptomics, proteomics and metabolomics) have highlighted that categorizing microglia into simplistic dichotomies like "good or bad" or "M1 or M2" does not adequately capture the full spectrum of microglial states across disease settings, and that updated nomenclatures and terminologies are warranted [113]. We agree with this principle, and to that extent, in assessing the role of microglia in pre-clinical TBI, we use the term Trauma Associated Microglia (TAM) to characterize the unique functionality of microglia in TBI versus other disease contexts (Fig. 1).

In healthy environments, microglia surveil their environment and can aid in synaptic remodeling, cell survival, and controlled phagocytosis removing CNS debris and waste products [114–116]. Morphologically, microglia are described as ‘ramified’ in their normal homeostatic state, and transform to a spectrum of morphologies during disease. In models of TBI, a dynamic range of TAMs are observed at both acute and chronic timepoints, initially clearing necrotic debris by adopting phagocytic states in response to ATP mediated purinergic signaling [117–119]. In the days and weeks following TBI, microglial cells undertake various functions, including, but not limited to, initiating immune responses, synaptic engulfment and clearance phagocytosis, influencing neuronal activity through bidirectional synaptic contact with neurons, and the potential to promote neurogenesis [120–123]. This dynamic functionality is generally delineated by the expression of Cd11b, CD68, CD86 and MHCII markers and accompanied by the release of proinflammatory mediators like TNF-α, IL-1β, NOX, IFNs or alternatively the expression of CD206 and Arg-1 markers and the release of anti-inflammatory factors including IL-10 and TGFβ [117, 121, 124–129]. Mechanistically, this functionality is determined by immune triggers that signal microglia to undergo morphological alterations resulting in highly activated states where they release soluble factors [118, 130]. Specifically, microglia are shown to respond to DAMPs activating PRRs, with TBI upregulating HMGB1, TLR2, TLR4 and NF-κB [31, 131–133]. Moreover, TLR2- and TLR4-deficient mice display decreased levels of TNF-α, IL-1, IL-6 and NF-κB signaling, evidencing their role in the TBI-induced pro-inflammatory response [134, 135]. In addition to TLRs, purinergic receptors P2Y6, P2Y12, and P2X4, detect ATP released from damaged cells and influence microglial responses after TBI [118, 119, 136]. Alongside the production of cytokines and chemokines, TAM also adopt functional states that promote oxidative stress through chronic NOX2 activation [137, 138]. In recent times, pharmacological depletion of microglia in the subacute and chronic timepoints after TBI is shown to be neuroprotective and improves outcomes [127, 139], underscoring the key role they play in deleterious TBI-induced neuroinflammatory responses.

Similar to microglia, astrocytes undergo morphological, molecular, and functional remodeling, with their classification now determined by the sum of their multi-factorial impact in context specific pathological settings [140]. In healthy contexts, astrocytes contribute to immune signaling, synaptogenesis regulation, BBB formation and maintenance, neurotransmitter recycling, ion and water homeostasis, and blood flow control [141–145]. In TBI, astrocytes are best known for the formation of the glial scar as a protective mechanism to limit secondary injury and promote regeneration [104, 146–149]. Astrogliosis is categorized by increased GFAP^+^ and vimentin across TBI models, yet away from the glial scar, the morphological significance of this gliosis is temporally and spatially dynamic [14]. They contribute to brain edema after injury [150–152], and contain decreased expression of GLT-1, GLAST and EAAT1/2, evidencing their role in TBI-induced glutamate dysregulation [153–156]. They also contribute directly to immune responses through DAMP/TLRs signaling as well as proinflammatory cytokine and chronic complement production after TBI [157–160]. Recent advances in high throughput sequencing and ‘omic analysis has generated datasets to begin to unravel the context specific nature of astrocytes in TBI [127, 161–164], yet these studies are still in early stages, especially when considering the range of heterogeneous immune responses that underpin the hallmark characteristics of TBI (Fig. 1). Furthermore, these responses often involve more than just a single cell type, exhibiting bidirectional communication, as evidenced by HMGB1 release from necrotic neurons, activating microglial TLR4, and subsequently increasing levels of astrocytic aquaporin-4 (AQP4), influencing BBB dynamics after TBI [31]. Indeed, this communication is now being explored in disease settings, with microglial release of C1q, IL1β and TNFα shown to transform astrocytes into neurotoxic states via their secretion of lipids contained in APOE and APOJ [165–167]. Blockade of this interaction is beneficial after stroke [168], suggesting studies of this pathway in TBI may yield promising targets to reduce inflammation mediated cell death pathways.

### Adaptive immune responses

Peripheral interactions and cell specific processes play important roles in the overall neuroinflammatory response in TBI. Temporally, within minutes an initial wave of neutrophils crosses the BBB to phagocytose injured tissue, followed by potent chemoattractant signals that encourage the migration and infiltration of peripheral macrophages and lymphocytes (Fig. 1). The temporal series of these cellular events within the brain parenchyma has been extensively reviewed elsewhere [169, 170]. Within the scope of this review, we would like to discuss the contribution of the adaptive immune response in relation to its interaction at meningeal barriers, including the recently discovered immune rich lymphatic vessel interface.

## Meningeal structures and barriers

The meninges, essential components of the CNS's protective and functional architecture, are composed of three distinct layers: the innermost pia mater, the intermediate arachnoid, and the outer dura mater. Structurally, the dura predominantly comprises collagen fibers that anchor it to the skull, as well as thin layer of fibroblasts demarcating the dura from the arachnoid [171]. In addition, the dura contains fenestrated blood vessels and lymphatics that form a connection with the periphery [171–176]. Beneath the dura lies the arachnoid barrier layer, composed of an outer layer of epithelial-like cells interconnected by tight junctions [177, 178]. It is this barrier that serves as a key component of the blood-CSF barrier, restricting the movement of molecules from the dura to the subarachnoid space [171, 175, 179]. The subarachnoid space plays host to vasculature networks and immune cells, with arachnoid trabeculae consisting of flattened fibroblasts-like cells connecting the arachnoid to the pia [180, 181]. CSF flows within this space and provides brain nourishment and buoyancy, as well as waste removal via venous flow and resorptive transport in the choreoid plexus [182, 183]. Tightly adhered to the brain, the pia mater is composed of a thin layer of fibroblasts, followed by a basement membrane that separates it from the underlying glia limitans. Collectively the arachnoid and pia are commonly referred to as the leptomeninges [184], within which the non-fenestrated vasculature contains tight junctions to form the leptomeningeal barrier [184–187]. Lastly, the glial limitans is constituted by astrocytic end feet processes to form a boundary between the brain parenchyma and the pia.

## Meningeal lymphatic vessels (mLVs)

The presence of lymphatic vasculature within the meninges, though hinted at and often misconstrued in various anatomical accounts in the seventeenth century [188], was first visually depicted in the late eighteenth century by the anatomist Paolo Mascagni [189]. However, subsequent to this, knowledge of these lymphatic vessels lapsed into relative obscurity until the mid-twentieth century [190]. Its 'rediscovery' in modern times in both mice [173, 175] and humans [174, 176] had led to a resurgent wave of interest within the neurosciences, challenging the long-lasting dogma of the “immune-privileged” brain. Anatomically, healthy meningeal lymphatic vessels (mLVs) are found alongside dural sinuses, arteries and veins, including the superior sagittal sinus, transverse sinus, sigmoid sinus, retroglenoid vein, rostral rhinal vein, middle meningeal artery, and pterygopalatine artery [173, 175, 191–194]. Insights into the mechanistic development of lymphatic vessels is largely drawn from studies of networks in peripheral tissues. In mice, lymphatic vasculature (LV) formation is predominantly venous-derived, originating from the cardinal vein during embryogenesis. At embryonic day 9.5 (E9.5), a subpopulation of venous endothelial cells express Sox18 (a SRY-related HMG-box transcription factor), which activates prospero homeobox 1 (PROX1) [195–198]. This PROX1 activation induces specific lymphatic endothelial cell (LEC) gene expression, and inhibits blood endothelial cell-specific genes by binding to the nuclear receptors COUP-TFII [199–202]. Indeed, Prox1^−/−^ endothelial cells fail to express LEC markers, instead retaining blood vascular endothelial properties [195], evidencing their role as a master regulator of lymphatic identity. Ultimately Sox18-PROX1 activation initiates the acquisition of LEC properties for the subsequent creation of lymph sacs and lymphatic vessel networks [203, 204]. Additional lymphangiogenic mediators include lymphatic vessel endothelial hyaluronan receptor 1 (LYVE-1), which increases the expression of the platelet aggregating protein podoplanin (PDPN), in LECs at E12.5. Vascular endothelial growth factor C (VEGF-C) activation of vascular endothelial growth factor receptor-3 (VEGFR-3) is also essential for the sprouting of LVs from the embryonic veins [205, 206], allowing for the ability of LECs to migrate and form the lymphatic sacs, visible at E12.5 [207]. Buds and sprouts progress until E14.5, by which time the lymphatic development stage is completed [208, 209].

In contrast to the embryonic formation of peripheral LV, the development of the intracranial mLV occurs postnatally, in a VEGF-C dependent manner [210]. Starting at the base of the skull LEC sprouting begins at birth, postnatal P0. Temporally, lymphangiogenesis continues in a characteristic pattern alongside veins, arteries, and cranial nerves during the first weeks. The mLVs appear at the cribriform plate at P2, middle meningeal artery after P4, and grow into the transverse sinus at P8 [210]. Between P13 and P20, lymphatic vessels expand along the transverse sinus appearing at the confluence of sinuses at P16 [191, 210], before extending the length of the superior sagittal sinus toward the olfactory bulb by P20 [191]. At this time, the mLVs become functional, draining content from the CNS into the peripheral lymphatic system at the base of the skull, with the mLV network fully developed at approximately P28 [175, 191, 192, 210–212].

In terms of functionality, mLVs can be categorized into thin-walled “initial” lymphatic vessels, and the larger “collecting” vessels. In mice, initial lymphatic vessels are highly permeable and are located dorsally near both the transverse and superior sagittal sinus. Initial lymphatics lack smooth muscle cell (SMC) coverage, and are composed of discontinuous button-like LEC junctions [173, 175, 213]. These button-shaped junctions consist of tight junction- and adherens associated proteins, that attach adjoining LECs at the base of interdigitating flaps between cells [214–216]. This flap-like overlap creates mini-valves that permit the entry of interstitial fluid, macromolecules, soluble antigens and immune cells including antigen presenting cells (APCs) [215–221]. In contrast, collecting lymphatic vessels at the base of the skull are surrounded by SMCs to help propel fluid movement and contain tight continuous zipper-like connections and secondary intraluminal valves [217, 220]. This makes them virtually impermeable, facilitating unidirectional drainage, whilst simultaneously preventing lymphatic backflow [222, 223]. In both mice and humans, collecting meningeal lymphatic vessels extend along the jugular vein, exit the skull through various foramina, before merging with peripheral collecting lymphatics that predominantly drain into the deep cervical lymph nodes (dCLNs) [173, 174, 192, 210, 224–226]. On the journey between initial to collecting lymphatics, fluid transitions into specialized pre-collecting lymphatic vessels. Located at the basal foramina, they exhibit features of both initial and collecting vessels, as they lack SMC, but contain one-way valves and a mix of both button- and zipper-like junctions [192, 227]. These findings emphasize the distinct morphological characteristics of various mLV subsets, providing insight into their specialized roles in immune cell and CSF waste clearance cascades from the CNS to the periphery.

## The (previously missing) link from the brain to the periphery

The 2015 multi-group discovery of mLVs in the dura mater, evidenced a role for these vessels as an initial collection site for CSF before its drainage to the dCLNs. Physically, the arachnoid barrier delineates the CSF contained in the subarachnoid space from the dura mater; however, the mechanism by which the CSF carrying waste products and immune signals from the brain reaches these mLVs in the dura was unclear. It has previously been suggested that arachnoid granulations, which are protrusions of the arachnoid mater into the venous sinuses of the dura mater, enabled drainage of CSF directly to the bloodstream [228], but tracers administrated into the CNS or CSF can drain directly to the dCLNs, demonstrating that there must be a direct route from the CSF to the dura that bypasses the bloodstream [194]. In 2024, a pioneering study identified arachnoid mater cuffs around the bridging veins that connect the subarachnoid space to the dura [229]. This finding was made by experiments that involve injecting tracers into the intracisternal magna (i.c.m) and observing their transport. The experiments revealed that the i.c.m. tracers were being transported into the dura before they had reached either the blood or the dCLNs, indicating that the drainage occurred directly from the CSF into the dura before being drained out by lymphatic vessels. This connection between the dura mater and the subarachnoid space consists of bridging veins that create discontinuities in the arachnoid barrier, forming structures termed arachnoid cuff exit (ACE) points. ACE points are present in humans and can work bi-directionally, enabling molecules and immune cells to enter from the dura mater into the brain [229]. This may explain how cytokine responses from meningeal immune cells influence brain function, a discovery that will shape the way we think about neuroinflammation in future TBI studies.

## The immunological environment within the meninges

It is now recognized that the meningeal layers act as vital immunological reservoirs, hosting an array of adaptive immune cells that first arrive in development via blood vessels or calvarium bone marrow that connects the dura via specialized vascular channels [211, 230–235]. Collectively, the meningeal layers contain lymphoid cells, macrophages, mast cells, eosinophils, dendritic cells (type 1 and type 2 classical dendritic cells, plasmacytoid dendritic cells and migratory dendritic cells), neutrophils, innate lymphoid cells (ILCs), natural killer cells, plasma cells, B cells (immature and mature) and T cells (CD4, CD8 and T cell receptor gamma/delta (TCRγδ)) [186, 211, 231, 236–240]. Proportionally, the distribution of these cells varies across layers, with the dura largely thought to contain a higher diversity and frequency of immune cells versus the leptomeningeal layer [186]. This may be attributed to the need for tissue specific support, with the niche environment around dural sinuses containing innate lymphoid cells, macrophages, T cells, B cells and plasma cells [234, 241]. In leptomeningeal layers, macrophages, CD4, and CD8 cells, are found in large part around blood vessels in the CSF containing subarachnoid space [242–245].

## Meningeal macrophages

Macrophages are heterogeneous groups of cells that carry out distinct functions depending on their location and phenotype [246]. Meningeal macrophages belong to a specialized group called border associated macrophages (BAMs), originating from yolk sac CD206^+^ myeloid progenitor cells [230]. In the adult, leptomeningeal BAMs can be transcriptionally delineated by CD206, Lyve1, P2rx7, and Egfl7 expression [186, 236]. In contrast, adult dural BAMs differ to their leptomeningeal counterparts as they lack Lyve-1 expression, and can be transcriptionally divided into subgroups based on major histocompatibility complex II (MHCII) expression [247, 248]. This may represent divergent roles under homeostatic or disease conditions, with MHCII^Hi^ BAMs displaying enhanced levels of CCR2, whereas MHCII^low^ BAMs contain gene signatures that include Clec4n, Clec10a and Folr2 expression [186, 211, 246, 249]. Of note, a determining factor that drives postnatal MHCII high or low differentiation, is the *fms* intronic regulatory element (FIRE), a highly conserved super enhancer of the colony stimulating factor 1 receptor (CSF1R) and its downstream signaling [250, 251]. This may be pertinent to consider in studies examining microglial driven inflammation in CSF1R deletion models (or via Pexidartinib (PLX) inhibition), as the effect of CSF1R in modulating facets of meningeal immunity has yet to be fully investigated. In healthy settings the functional role of BAMs is largely unknown, however evidence suggests they can act as sentinels in anti-viral defense. Following lymphocytic choriomeningitis virus infection (LCMV), BAMs become rapidly activated, acquire the viral antigen and trigger engagement by infiltrating cytotoxic T lymphocytes that are essential to resolve the infection [252]. Further, the type-1 interferon-Stat1 anti-viral pathway is induced in BAMs, blocking further LCMV infection [253]. Transcranial delivery of PLX3397 or PLX5622 to specifically deplete BAMs resulted in the death of LCMV-infected mice [253], emphasizing the importance of further investigation into BAMs in CNS disease models that contain inflammatory components within their etiology.

## Border associated dendritic cells

**Fig. 2:**
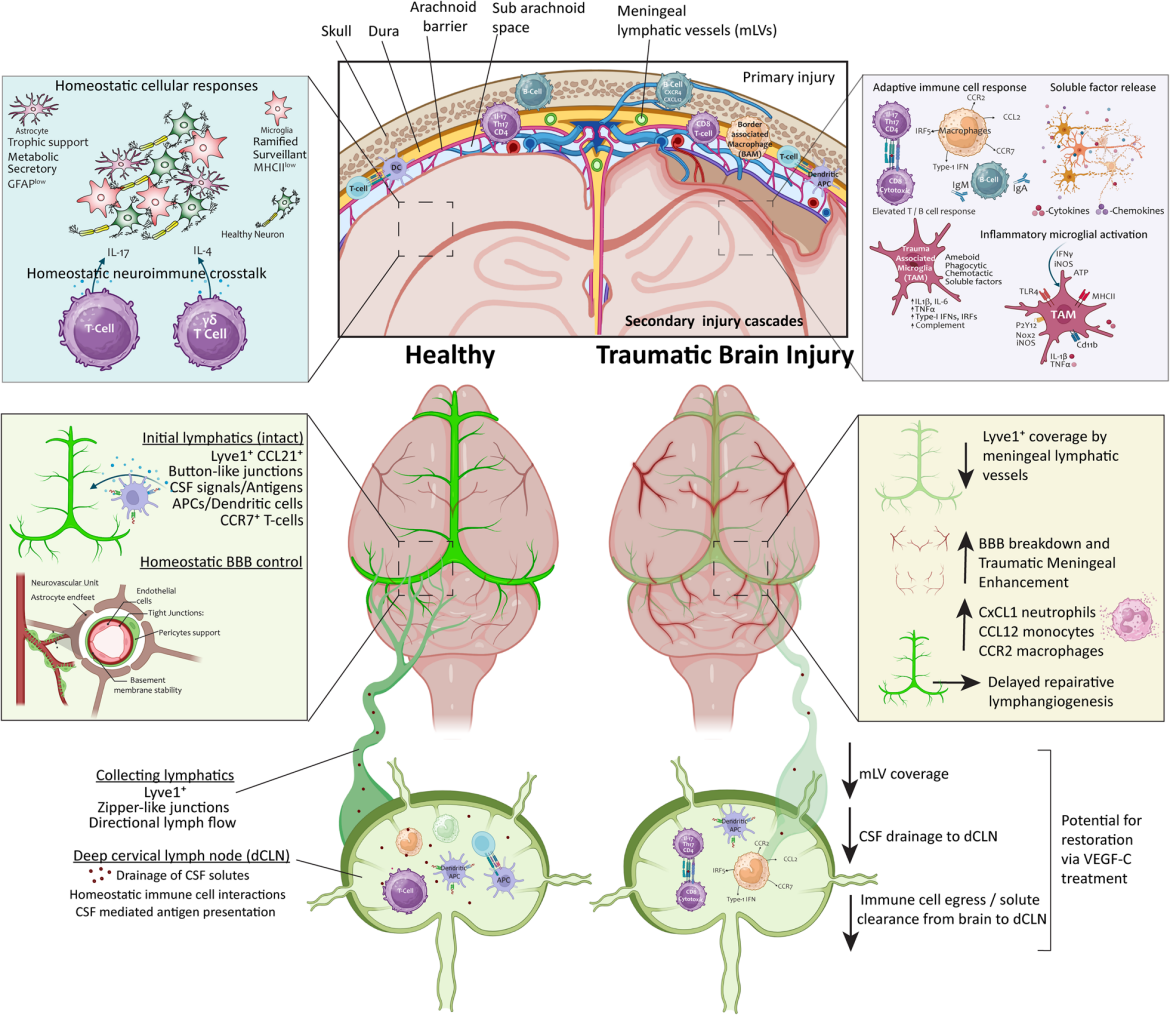
The meningeal neuroimmune interface influencing inflammatory responses in TBI. Simplified schematic of the meningeal interface in healthy and injured settings. In homeostatic conditions (left), border-associated immune cells may synergistically support brain environments through cytokine secretion (IL-4, IL-17), which can directly influence neurons. A network of initial and collecting mLVs expressing Lyve1, drain CSF solutes/molecules to the dCLNs. Immune cell drainage of dendritic and T cells into the dCLNs occurs via chemokine gradients (E.g. CCR7 expressing cells migrating towards its ligands CCL19 and CCL21). TBI (right) causes BBB breakdown and mLV dysfunction, resulting in reduced Lyve^+^ mLVs, and impaired drainage of solutes to the dCLNs. This TBI-induced lymphatic dysfunction and immune activation at the meningeal interface, exacerbates neuroinflammation by resident CNS cells within the brain parenchyma

Dendritic cells (DCs) are professional APCs that play key roles in immune surveillance in both peripheral and meningeal layers [254]. DCs in the meninges and choroid plexus arise from circulating and skull bone marrow precursor-DCs, which transform into conventional dendritic cells (cDCs) at these sites [233, 255, 256]. Investigations into their trafficking ability, revealed that peripheral DCs can migrate into the CNS across barrier sites via the C–C motif chemokine receptor 2, chemokine ligand 2 (CCR2-CCL2) signaling pathway [257]. This migration can be bi-directional, with DCs observed in the cervical and auxiliary lymph nodes 3–7 days after their injection into the CSF or brain parenchyma [258–260]. Meningeal mediated migratory drainage of DC occurs via initial mLVs in a CCR7 dependent manner. CCR7 is a potent receptor of DC migration toward the ligands CCL19 and CCL21, which are highly expressed by lymphatic vessels (Fig. 2). In pathological states, DCs recognize and capture antigens, upregulate CCR7 and migrate to initial mLVs before draining into the dCLNs where they can activate antigen-specific T-cell inflammatory responses [173, 225, 239, 261]. Indeed, dural and cribriform plate mLVs express CCL21, facilitating a gradient for DC migration and drainage into peripheral lymph nodes [175, 211, 225, 262]. Additionally, dural fibroblast-like cells express CCL19, providing further stimulus for CCR7 mediated signaling within the meninges; however, the role of these fibroblasts in relation to CNS lymphatic drainage is yet to be elucidated [211]. Collectively, DC populations within the meninges and CSF may contain both parenchymal infiltrating DCs as well as migratory draining DCs in transit to lymph nodes, suggesting that the holistic neuroinflammatory environment unique to specific disease states influences DC capacity, drainage and antigen presentation.

## Border associated T cells

The interplay between the innate CNS and adaptive immune response contributes to neuroinflammation within disease contexts. T cells are found in the meningeal space, with populations identified at dural and cribriform lymphatic sites [173, 192, 262]. Initial evidence of T cell migration into dCLNs was noted 12–48 h after their CNS injection into healthy or lesioned mice [263], with subsequent lymphatic ablation studies offering extensive characterization of T cell migration pathways via dural mLVs [225]. Mechanistically, drainage of T cells into the dCLNs via mLVs occurs in a CCR7 dependent manner [225], a process similar to that seen in inflammatory-induced peripheral T cell migration [264, 265]. Indeed, CCR7 expressing T cells are found in human CSF, most of which are thought to be central memory T cells conducting immune surveillance within the healthy subarachnoid space [266, 267]. While trafficking to dCLNs may reduce meningeal T cell pools, they are replenished by blood circulating T cells that enter the brain via the dural sinuses, and adopt tissue-resident phenotypes [211, 231]. Indeed, evidence shows elevated densities of APCs and T cells at dural sinuses, serving as an active site for APCs to acquire blood- or brain-borne pathological antigens, presenting them to local T cells [211]. Functionally, the role of a well-regulated adaptive immune response is paramount, as CD4^+^ and TCRγδ cells can influence CNS neuronal, glial, and homeostatic activities through cytokine secretions including IL-4, IL-17 and IFNγ [231, 268–272]. Furthermore, mice lacking functional T cells, and consequently deficient T cell drainage to dCLNs, have notable deficits in exploratory, social, and cognitive behaviors. [268, 273, 274]. These deficits are reversed following adoptive transfer of T cells or secondary lymphoid organ lymphocytes, further evidencing their important role in the regulation of CNS behaviors [268, 274, 275].

## Border associated B cells

B cells play versatile roles within the adaptive immune response. Known predominantly for their antibody production and as precursors to antibody-producing plasma cells, they also function as APCs, and producers of proinflammatory cytokines and chemokines (reviewed in [276]). In humans, the expression of B cells and plasma cells is sparse within the CNS parenchyma [277, 278], however they are abundantly located in the meninges [277], specifically the dural layer [279]. In mice, the identification of meningeal B220^+^ CD11c^−^ B cells was first reported within the dural mLVs [173], with subsequent single-cell sequencing studies identifying a diverse range of progenitor, early and resident B cell populations [234, 279–281]. Constitutively, B cells represent ~ 15–30% of all CD45^hi^ cells within the dural meninges, encompassing multiple stages of B cell development from pro-B to mature B cells [234, 279]. Meningeal B cells derive locally from the calvaria bone marrow niche before migrating to the meninges through specialized skull vascular channels [234]. Following this calvarial–meningeal migration, B cells complete their development locally, with factors such as CXCL12 and CXCR4 critical for their survival and differentiation [234]. IL-7 has also been implicated in supporting B cell development, alongside other niche factors in the meninges including the CNS specific antigen myelin oligodendrocyte glycoprotein [281]. For the most part, the majority of mature B cells in the CNS are naïve IgM^+^ cells, with small numbers of IgA^+^ B cells [234, 279]. In terms of migration, B cells have the capacity to drain to the dCLNs via mLV in a similar fashion to their T cell counterparts. Plasma cells (IgA^+^) are also found in the meninges, with a portion of this population derived from gut plasma cells [282]. This meningeal plasma cell expression is age dependent, with young mice displaying IgA+ cells, switching to an IgG^+^ and IgM^+^ plasma cell phenotype in aged mice [234].

## Neuroimaging of meningeal disruption in clinical TBI

Neuroimaging provides valuable data to assist in patient evaluation and diagnosis following TBI. Common imaging modalities include conventional non-contrast computed tomography (CT) and magnetic resonance imaging (MRI), often used in the clinical work-up for injury indications in patients with GCS < 13 [283]. CT scans provide initial class I assessment of injury extent (lesion, fracture), extradural, subdural or intracranial hemorrhage, traumatic subarachnoid hemorrhage and ventricular abnormalities [284, 285]. This data is beneficial for diagnosing the mechanically induced primary trauma, yet sophisticated imaging techniques are necessary to detect subtle injuries to the brain structure and secondary injury processes. Enhanced imaging of dysregulated secondary injury cascades including glucose metabolism (functional MRI), diffuse axonal injury (diffusion tensor imaging, DTI), protein accumulation and neuroinflammation (radiotracer positron emission tomography, PET) could lead to the discovery of new imaging biomarkers and therapeutics [286].

Advancements in MRI using contrast agents (such as gadolinium) have improved the specificity of diagnostic images, allowing for identification of novel biomarkers and insights into disease progression [287, 288]. Ordinarily gadolinium is unable to cross the blood–brain barrier, and therefore acts as a surrogate for leakage of proteins and other macromolecules when detected outside of the vasculature [289]. Gadolinium post-contrast fluid-attenuated inversion recovery (FLAIR)-MRI merges a high intensity of T2 weighting with the attenuation of cerebrospinal fluid (CSF) signal. This allows for the detection of leakage of the gadolinium contrast agent across damaged barriers into the CSF space, and highlights hyperintense regions at CSF adjacent borders. This is relevant in the context of TBI, as vascular injury can occur in the parenchyma and vessels traversing the meninges [290]. Meningeal arteries and veins in the dura and subarachnoid space are particularly vulnerable to the primary impact of TBI. Indeed, recent findings suggest that Traumatic Meningeal Enhancement (TME), associated with meningeal injury and inflammation, is a novel biomarker observed by FLAIR-MRI in patients following TBI (Table 2).

**Table 2:** Imaging of meningeal-brain border disruption in clinical TBI

TBI Severity	Age (IQR)	MRI	Image time (h/d)	Result	N (%)	Meningeal enhancement observations	Refs.
mTBI GCS = 15	THINC (Age undisclosed)	FLAIR	Within 48 h	ME	69/142 (48.6%)	Focal enhancement	[119] 2014
mTBI GCS ≥ 13	59.8 (26–84)	FLAIR	3.2 d (0.2–14 d)	TME	32/54 (59%)	Diffuse (12/32), Localized (8/32), Falx (12/32)	[291] 2014
mTBI GCS = 15	48 (41–55)	FLAIR	5.4 h (3.6–5.3)	TME	10/22 (45%)	Diffuse (4/10), Localized (3/10), Falx (3/10)	[292] 2017
mTBI GCS > 12	42 (28–55) THINC	FLAIR	11.6 h (4.9–20.2 h), Temporal follow-ups	TME	104/209 (50%)	ME resolved in 79 patients (76%) at 22d (7–37 d). Chronic ME in 17% of patients at 87 d (72–103 d)	[293] 2018
mTBI GCS ≥ 13	39.8 (22.7–57.8) THINC	FLAIR	21 h (14–26 h)	TME	17 TME+ 13 TME-	76 DEGs in TME + vs TME- ↑ IgA ↑FCαR, ↑MCTP2, ↑ GPR27, ↓CD79A	[294] 2017
mTBI GCS ≥ 13	49 (35–62) THINC	FLAIR	6.07 h (4.3–19.6 h)	TME	9/25 (36%)	Conspicuity of TME is higher on FLAIR MRI than on post-contrast T1WI	[295] 2020
Mild 7, Mod 19, Severe 4	46.4 (SD 16.5)	FLAIR	within 48h	TME	16/30 (53%)	Of 16 with TME, 10 (63%) resolved 1-year follow-up, 6 TME persisted	[296] 2020
mTBI GCS = 15	39.8 (22.7–57.8) THINC	FLAIR	19 h (5.9–44 h)	TME	12/36 (33%)	Regions- 11 Falx, 6 Vertex, 5 Frontal, 4 Temporal, 3 Occipital, 1 Cerebellar	[289] 2020
mTBI GCS = 14–15	57 (41–67) THINC	FLAIR	4.3 h SD ± 0.87	TME, ECSAS	44/75(59%), 2^nd^scan 23/32 (72%)	18/32 (56%) positive for ECSAS	[289] 2020
Fatal vertical falls	Data not provided	Dural Tissue	24 h for Histology	+ IHC	4 samples	↑VEGFR2 in blood vessels in injured dura mater	[297] 2020
Moderate-to-severe	39.2 (28–56)	Dural Tissue (1 m^2^)	Resected from de-compressive craniectomy	Flow, ex vivo assays	5 males, 1 female	ILC1: CD45^+^Lin^−^CD127^+^CD161^+^ NKp44^+^, produce IFNγ; ILC2: CD45^+^Lin^−^ CD127^+^ GATA3^+^ CRTH2^+^, produce IL-5/IL-13; ILC3: CD45^+^ Lin^−^ CD127^+^ RORγt^+^AhR^+^, produce IL-17	[298] 2021

mTBI: mild traumatic brain injury; GCS: Glasgow Coma Scale; MRI: magnetic resonance imaging; IQR: interquartile range; FLAIR: fluid-attenuated inversion recovery; ME: Meningeal enhancement; TME: traumatic meningeal enhancement; THINC: Traumatic Head Injury Neuroimaging Classification study (NCT01132937); DEGs: differentially expressed genes; ECSAS: extravasation of contrast into the subarachnoid space; HARM: hyperintense acute reperfusion marker; ILCs: innate lymphoid cells

Although meningeal enhancement has been observed in neuroinflammatory contexts and in neurological disease [299–301], its identification in TBI is a relatively recent development (Table 2). In 2014, Roth and colleagues first reported focal enhancement of the meninges in approximately 50% of patients following a mild TBI (mTBI) [119]. A temporal follow up study identified similar TBI induced TME positive patients (50%) [293], of which 76% displayed resolution by approximately 22 days post-injury; however, 17% had persistent TME for several months (72 to 103 days post injury) [293]. This TME time course may be associated with, and influenced by, injury severity, with TME detected in moderate to severe TBI patients at 1-year post injury [296]. Collectively, these likely represent chronic inflammatory or other secondary injury cascades occurring within this compartment in the weeks, months, and years post-injury. Anatomically, TME positive mTBI patients display thick linear meningeal enhancement of the dura, including diffuse and localized convexity patterns, as well as enhancement of the falx cerebri [119, 289, 291, 292]. As the volume of data and studies expand, the diagnostic value of positive TME findings has become more apparent. TME signatures have been shown to be associated with loss of consciousness, implying clinically significant head injury [291], and FLAIR imaging protocol demonstrate superiority in detecting trauma-related abnormalities not visible on CT scans, distinguishing between acute trauma from nonspecific conditions [292]. Specifically, FLAIR protocols outperform T1W1 post-contrast sequence in identifying the presence or absence of TME, where T1W1 failed to show it in 38% of patients where it was readily shown using FLAIR [295]. To gain insights into biological mechanisms associated with TBI-induced meningeal enhancement, transcriptomic analysis from patients with TME identified 76 differentially expressed genes, including increases in IgA, FCαR, MCTP2, GPR2, and decreases in CD79A [294]. Furthermore, innate lymphoid cells (ILCs) are found in the dura and CSF of TBI patients, suggesting meningeal immune interactions [298]. Increases in VEGF2 expression in meningeal vasculature [297], suggests regenerative capacity of dural vessels; however, this finding should be repeated in subjects with clinical classification data, for meaningful translational interpretation.

## Meningeal lymphatic dysfunction after TBI

Neuroinflammation, characterized by the intricate interplay between innate and adaptive immune responses, contributes to the pathophysiology of TBI (Fig. 1). A fine balance exists whereby acute activation of neuroimmune cells may be a beneficial physiological response to promote injury resolution, in contrast to aberrant neuroimmune activation that may manifest toward deleterious chronic outcomes. Identification of the immune rich lymphatic system at the meningeal barrier interface, has triggered a new area of previously unexplored research, investigating the contribution of this neuroimmune crosstalk to TBI-induced neuroinflammatory responses (Table 3) In 2020, Bolte et al. reported seminal findings evidencing the temporal series (across seven timepoints) of meningeal lymphatic disruptions in a closed head impact model of TBI. Here, they detail mLV drainage of CSF molecules to the dCLNs is impaired as early as 2 h, and persists for at least 1-month, following injury [302]. This impaired drainage was associated with changes to lymphatic vasculature, with a decrease in Lyve-1 expression observed within the first 2 to 24 h post-injury [302]. Interestingly, mLVs demonstrated a capacity for regenerative lymphangiogenesis, evidenced by the spontaneous increase of Lyve-1 coverage and complexity in meningeal whole mount preparations 1–2 weeks post injury [302]. Photothrombotic vascular damage to the dura mater and pia mater causes immediate vascular degeneration followed by revascularization at 7d post-injury [297], suggesting that changes in mLVs may occur in parallel with regenerative endothelial angiogenesis at dural sites (Fig. 2). However, across the spectrum of TBI, the extent of this naturally occurring vascular and lymphangiogenic responses varies upon injury type and severity. Lyve-1^+^ mLV coverage/expression is decreased at 3d (mice, impact 1.9atm) [303] and 7d (rats, impact 2.6 atm) [304] after lateral fluid percussion injury (FPI). MRI of a closed-head impact model of engineered rotational acceleration (CHIMERA) shows meningeal enhancement 7d after single and repeat injury, reflecting clinical observations [305]. Controlled cortical impact (CCI) in mice presents mixed findings: Lyve-1^+^ morphology is increased at 7 d (impact 1.5 mm) [306], with contrasting studies showing decreases at 3 d (impact 2 mm) [307], and 1 month (impact undisclosed) [308], highlighting the nuanced and variable nature of spontaneous lymphangiogenic responses across varied mechanisms of impact and injury parameters. Regardless, if plasticity of the lymphatic network can be induced, it may be beneficial to improve outcomes (Fig. 2). Indeed, across models of TBI, overexpression of VEGF-C drives lymphangiogenesis, enhances functional drainage and improves injury induced neurological deficits, including those seen in aged animals [303, 304, 306, 308–310].

**Table 3:** Meningeal insights in pre-clinical models of traumatic brain injury

TBI model	Animal/Age/Sex	Interval time	Tissue/Cell type	Injury-induced meningeal signature	Refs
Thinned skull, manual blade compression mTBI	Male, Female C57Bl/6J mice (8–12 wks)	6 h, 1 d, 4 d	Whole mount meninges, QPCR analysis	At 6 h, 1 d ↑Cxcl1 (neutrophils), ↑Ccl2, Ccl12 (CCR2^hi^monocyte) ↑Cxcl10, Il1β At 1 d, 4 d↑ vascular leakage At 4 d ↑wound-healing CD206^+^ Lyve-1^+^ macrophages	[293] 2018
Photo-thrombotic injury	C57Bl/6J mice (8–10 wks)	1 d, 7 d,	Whole mount dura and pia, Dura–RNA & protein	Dura mater revascularization by 7 d post injury	[297] 2020
Adapted CCI hit and run (closed head, 2 mm, 5.2 m/s, dwell time 100 ms)	Male, Female C57Bl/6J mice (8–10 wks)	2 h, 1 d, 4 d, 1 w, 2 w, 1 m, 2 m	Whole mount meninges & dCLN	At 2 h, 1 d, 4 d, 1 w, 2 w, 1 m ↓ MLVs drainage to dCLN. At 2 m drainage restored At 1 w, 2 w↑Lyve1 coverage, at 1 m, 2 m ↑loops/complexity	[309] 2020
Thinned skull, manual blade compression mTBI	Male, Female C57Bl/6J mice (8–12 wks)	1 d, 3 d, 4 d,5 d, 7 d	Whole mount meninges	At 1 d ↓endothelial vascular integrity. Revascularization at 3 d, 5 d,7 d. Inhibited by infection At 1 d, 3 d, 4 d and 7 d ↑ CD11b^+^, CD206^+^ myeloid cells	[321] 2021
CCI (3 mm, 3 m/s, dwell time 85 ms)	Male C57Bl/6J mice (9–10 wks)	1 d, 7 d, 1 year	Meninges, Meningeal ILCs (Innate lymphoid cells)	At 1,7d ↑ILC1-3. At 1y ↑ILC2/3 Metabolic dysregulation with ↓AMPKα1 at 1d *CCI + IL-33 (1μg) ↑pAMPKα	[298] 2021
CHIMERA (single × 1 & repeat × 4)	Male, Female C57Bl/6 mice (6–7 wks)	1 d, 7 d	Whole brain MRI	At 1d & 7d ↑ meningeal enhancement in both single and repeat impacts	[305] 2022
CCI (0.5 mm or 1 mm, 5 m/s, d well undisclosed)	Male C57Bl/6J mice (9–10 wks)	3 dpi, 6 wks	Dura mater–RNAseq, Whole mount meninges	3dpi - ↑CD45+ myeloid (B cell) & CD11b+ cells 6 wks - ↑Ccl8, Il1β, Ccl2 Ccl7 ↑immune pathways, including interferon gamma response	[316] 2022
CCI (2 mm, velocity undisclosed)	Male C57Bl/6 mice (10 wks)	3 d	Meningeal lymphatic endothelial cells (LECs)	Flow cytometry ↓ Lyve1 LECs Microarray ↑ DEGs involved in FCERI signaling, antibody-mediated complement, Inflammatory response	[307] 2022
Adapted CCI hit and run (closed head, 2 mm, 5.2 m/s, dwell time 100 ms)	Male C57Bl/6J mice (8–12 wks & 20 months)	7 d, 1.5 m	Whole mount meninges Meninges for bulk & scRNA-seq	At 7d, ↑DEG s related to macrophages, fibroblasts, and adaptive immune cells. ↑IFNβ, IRF5, IFNAR1 At 1.5m ↑ collagen, fibroblasts ↑T/B cell DEG. Aging amplified	[302] 2023
FPI (11°, 2.6 ± 0.16 atm)	Male, Sprague–Dawley rats	7 d	Whole mount meninges & dCLN	*FPI ↓MLVs drainage to dCLN ↓Lyve1, Prox1, Foxc2, VEGFR3 *FPI + VEGF-C/Ketoprofen/RA improves Lyve1 + dCLN drainage	[304] 2023
CCI (1.5 mm, 3m/s, d well time 120 ms)	(8–10 wks)	4 d, 7 d, 14 d	Whole mount meninges & dCLN	*CCI ↓MLVs drainage to dCLN 4 d, 7 d,14 d. ↑Lyve1 7 d *CCI + VEGF-C 156S&EVs@Gel restores dCLN drainage	[306] 2023
FPI (1.9 ± 0.2 atm)	Male, Female C57Bl/6 mice (8–10 wks)	3 d	Whole mount meninges & dCLN	*FPI ↓MLVs drainage to dCLN ↓Lyve1, ↓VEGFC/VEGFR3 *FPI + IL-33 (i.c.m, 20ng/μl, 5 μl), restores dCLN drainage, Lyve1, VEGFC/VEGFR3	[303] 2023
CCI (2 mm, 3.5 m/s, dwell time 500 ms)	Male C57Bl/6 mice (3 months)	28 d	Regrown cavity leptomeninges (pia, arachnoid)	Brain Fibroblasts (BFB 2–5) ↑Fmod (BFB2/3), ↑Dpp4 (BFB4), ↑Slc47a1 (BFB5) (drug transporter)	[184] 2023
CCI (depth, velocity, undisclosed)	Male C57Bl/6 mice (6–10 wks)	28 d	Whole mount meninges	*CCI ↓Lyve-1 area at 28d *CCI+ pVEGFC (i.v 50µg @1 d, 3 d, 5 d) restores Lyve1 (lymphangiogenesis)	[308] 2023
CCI (1.5 mm, 5.25 m/s, dwell time 100 ms)	Male C57Bl/6J mice (8–12 wks & 18–19 m)	7 d, 1 m	Meningeal bulk RNA-seq	At 7d ↑laminin, collagen and T-cell DEGs. ↑ TGFβ, IFNα. At 1 m ↑immunoglobulin production and B cell DEGs. ↑Ccr2 (young), IL-33 (aged) upstream regulators. ↑IFNα/β	[317] 2023

CCI: controlled cortical impact; FPI: fluid percussion injury; MLVs: meningeal lymphatic vessels; dCLN: deep cervical lymph nodes; meningeal lymphatic endothelial cells (LECs); CHIMERA: closed-head impact model of engineered rotational acceleration; ILCs: innate lymphoid cells

## The meningeal interface influencing TBI induced immunological responses

### TBI-induced meningeal damage

The meninges host a diverse array of immune cells, as previously outlined, yet the precise details of their locations (whether in the leptomeninges or dura) and trafficking patterns in TBI still remain largely unknown. Initial studies at the meningeal interface classified the nature of damage to the barrier itself, followed by the temporal response to direct meningeal injury. Acutely, closed head meningeal compression injury (achieved by a unique skull thinning model) is characterized by rapid meningeal macrophage cell death attributed to vascular leakage and the release of reactive oxygen species [119], and also causes secondary damage to the glial limitans and brain parenchyma within the first few hours [119, 293]. The initial injury is followed by neutrophil swarming into the meninges (within 1 h), that is essential for regeneration of the initially damaged glial limitans [119]. Elevated proinflammatory cytokines IL1α/IL1β are seen in the meninges at 6 h to 1 d, as well as the chemoattractants Cxcl1 (neutrophils) and Ccl2/Ccl12 (monocytes) [293]. Over the course of a week after injury, infiltrating myeloid cells (CX3CR1^lo–neg^CCR2^hi^ monocytes) scavenge dead cells at the meningeal lesion core, while wound-healing macrophages (CX3CR1^hi^CCR2^lo–neg^CD206^+^) proliferate along the lesion perimeter to promote angiogenesis through the clearance of fibrin and production of MMP-2 [293]. These studies at the level of the meninges evidenced the importance of the previously unexplored barrier immunity, setting the stage to address bi-directional interactions containing both extra-axial meningeal cascades and intra-axial brain parenchyma responses.

### Modulating meningeal lymphatics in TBI

Impairment studies shed more light on how the meningeal compartments influence CNS responses, with ablation of mLVs exacerbating resident glial cell (GFAP^+^, IBA1^+^) immunoreactivity, increasing complement, reducing neuronal health markers and which collectively influenced cognitive outcomes [309]. Furthermore, administration of the pro-lymphangiogenic VEGF-C to aged mice reduced brain gliosis and improved cognition. Integrating adaptive immunity, TBI induces the accumulation of Granzyme B^+^ CD8^+^ cytotoxic T cells, a response preceded by increases in IL-17-producing CD4^+^ T cells and IFNγ-producing CD4^+^ T cells [311]. This sequence is significant, given Th17 cell responses through IL-17 and IL-21, can enhance the cytotoxic capability of CD8^+^ T cells [311]. In K14-VEGFR3-Ig (TG) mice that have defective growth and lack mLVs alongside sclerotic dCLNs, a reduction in the perilesional infiltration of T-cells is observed [312]. Specifically, TBI-K14-VEGFR3-Ig mice show reduced levels of infiltrating CD4^+^ T cells, suggesting that trauma induced brain-derived antigens may be partially drained through the mLVs to the dCLNs to elicit Th-2 mediated responses [312]. Indeed, lymphatic vessels and LECs themselves play a direct chemoattractant role in the maturation of T cells, turning naive T cells into a memory-like subset of quiescent yet antigen-experienced CD8^+^ cells that can rapidly differentiate into an effector upon inflammatory antigenic challenge [313]. Dendritic antigen presenting cells can also survey the inflammatory CNS milieu, traverse the mLVs in a CCR7, CCL19 and CCL21 manner, and interact with T cells in the dCLNs. During trauma, DCs are elevated and influence post-injury immune responses [129, 259, 314, 315], yet this meningeal migration of DCs downstream activation of adaptive immune responses hasn’t been studied in the context of TBI.

### Insights from transcriptomic approaches

In order to gain a greater understanding of the cell specific responses that occur in the meninges after TBI, we and others have taken a transcriptomic approach [184, 302, 316, 317]. scRNA-seq shows increased meningeal macrophages, CD8^+^T cells, T helper cells Th2 and Th17, immature-mature B cells, dendritic cells, and fibroblasts in meningeal compartments at 1-week post injury [302]. Similarly, we identified time dependent alterations in the meningeal transcriptome. T-cells respond acutely, followed by chronic B-cell and immunoglobulin production in an age dependent manner, which may be regulated by upstream Type-1 IFNs, Ccr2 and IL-33 interactions [317]. Supporting this, meningeal macrophages display strong type-1 IFN signatures (elevated IFNβ and IRF5) and sub cluster into “inflammatory” and “resolution” classes [302]. Resolution macrophages express of antigen presentation-related genes (H2-Eb1, H2-Ab1, H2-Aa, Cd74) and anti-inflammatory genes Stab1, Nrros, and Dab2 which are involved in suppression of type I IFN responses. Conversely, meningeal inflammatory macrophages are defined by their expression of Ccr2 and chemotaxis genes Ccr7, Ccl22, and Ccl5.

### Future avenues for neuroimmune investigation

These seminal pre-clinical studies of the meningeal response after TBI prompt numerous inquiries for future investigation within the field of neurotrauma. Not only do the meninges harbor immune cells that contribute to a vast array of responses, the mLVs play a crucial role as the waste-removal system in draining CSF, ISF, and CNS-derived molecules from the brain parenchyma to the dCLNs. The effect of their damage caused by primary injury and how that influences removal of proteins such as Aβ and tau remain to be characterized. Additionally, stratification of the level of damage to the LVs, subsequent lymphangiogenic recovery, and the effect on the flow of CSF throughout the brain is still to be characterized. Transcriptomic studies suggest inflammatory molecules may regulate meningeal derived inflammatory responses, particularly chemokines. Indeed, modulation of the CCR2-CCL2 signaling axis in TBI has been shown to be beneficial [77, 318–320]. CCL2^−/−^ mice show delayed reductions in lesion size beginning at 28 days post injury. It might be postulated that this delayed response may be due to deficiency in meningeal mLV acutely, prior to lymphangiogenic recovery, in combination with altered CCL2 chronic immune responses. Indeed, CCR2^−/−^ mice show altered monocyte/macrophage influx and reduced crosstalk with innate cells, inhibiting the generation of type-1 IFN microglial responses after TBI, with pharmacological blockade of CCR2 improving cognitive outcomes at 28-day timepoints [318, 319]. Populations of macrophages after TBI were Mrc1^+^ Lyve1^+^ [319] suggesting microglial cross communication between meningeal cells, and as of now investigations into TBI mediated CCR2-CCL2 axis at the mLV immune interface are yet to be conducted, potentially warranting future investigation.

Alongside CCL2, CCL7 has been observed in the meninges single TBI [316]. CCR7 is expressed in a variety of peripheral immune cells including DCs and T-cells. Given CCR7^+^ immune cells migrate to the dCLNs via a CCL19 and CCL21 chemokine gradient (Brandum et al., 2021), suggests the potential DCs (that have phagocytosed TBI-induced antigens) and naïve T-cells migrate into LVs and dCLNs and present antigens to naïve CD8^+^ T-cells and CD4^+^ T-cells and activate them in response to TBI. Future studies investigating this potential using agonists/antagonists or conditional CCR7Ko models may be of interest to examine pathway influences neuroimmune outcomes and cognition in TBI, similar to that observed in 5xFAD models [239]. Most importantly, these questions may be influenced by factors like age, sex, and injury severity. Future studies should incorporate these variables to stratify the immune response at the meningeal interface following traumatic brain injury (TBI).

## Conclusions

TBI introduces a complex interplay between neuroinflammatory responses and immune dysregulation. We now understand that the meninges play a role in modulating this neuroinflammatory response, serving as the interface between the brain and the immune system; however, we still do not fully understand the mechanisms underlying prolonged neuroinflammation or how the temporal progression of damage to the meningeal lymphatic vessels influences cell trafficking and resolution. After TBI, immune cells (monocytes, macrophages, neutrophils) are available at the meningeal interface to immediately influence the brain, and infiltrating cells such as T and B cells can still be found in the brain and meningeal interface months following the injury. Does the presence of meningeal immune cells and the soluble factors released in response to trauma signal to, and amplify, the innate microglial responses? Are these cells being constantly trafficked into the brain, attracted by continuous damage signals? Do invading immune cells enter the CNS to mediate damage, but are unable to exit due to trauma-induced mLV dysfunction? Targeting the ability of immune cells to enter the CNS in response to injury, enhancing their ability to drain from the CNS, and gaining a better understanding of the crosstalk between the brain, meninges, and dCLN will allow us to harness the power of the immune system to enhance recovery from TBI.

## Data Availability

All data generated or analyzed during this manuscript are included in this article.

## Abbreviations

ACE: Arachnoid cuff exit
APC: Antigen-presenting cells
AQP4: Aquaporin-4
Arg: Arginase
ASC: Protein apoptosis associated speck-like protein
b2M: Beta-2-microglobuli
BAMs: Border associated macrophages
BBB: Blood brain barrier
BDNF: Brain-derived neurotrophic factor
C3: Complement component 3
CCI: Controlled cortical impact
CCL: C-C motif ligand
CCR: C-C chemokine receptor.
CCR2-CCL2: Chemokine receptor 2, chemokine ligand 2
CD: Cluster of differentiation
cDCs: Conventional dendritic cells
CHIMERA: Closed-head impact model of engineered rotational acceleration
COX: Cyclooxygenase
CR: Complement receptor
CRP: C-reactive protein
CSF: Cerebrospinal fluid
CSF1R: Colony stimulating factor 1 receptor
CT: Computed tomography
CXCL8: C-X-C motif chemokine ligand
DAMPS: Damage-associated molecular patterns
DC: Dendritic cells
dCLNs: Deep cervical lymph nodes
DEGs: Differentially expressed genes
DNAM: DNAX accessory molecule
ECSAS: Extravasation of contrast into the subarachnoid space
FBG: Fibrinogen
FIRE: Fms intronic regulatory element
FITC: Fluorescein isothiocyanate
FLAIR: Fluid-attenuated inversion recovery
FPI: Fluid percussion injury
GCS: Glasgow Coma Scale
GFAP: Glial fibrillary acidic protein
HARM: Hyperintense acute reperfusion marker
HLA: Human leukocyte antigen
HMGB1: High mobility group box 1
HSP: Heat-shock protein
i.c.m.: Intracisternal magna
i.v.: Intravenous
ICAM: Intracellular adhesion molecule
IFN: Interferon
IgG: Immunoglobulin G
IL: Interleukin
ILCs: Innate lymphoid cells
iNOS: Inducible nitric oxide synthase
IQR: Interquartile range
KIR: Killer-cell immunoglobulin-like receptors
LCMV: Lymphocytic choriomeningitis virus infection
LEC: Lymphatic endothelial cell
LV: Lymphatic vasculature
LYVE-1: Lymphatic vessel endothelial hyaluronan receptor 1
MCP: Monocyte chemoattractant protein
ME: Meningeal enhancement
MHCII: Major histocompatibility complex II
mLVs: Meningeal lymphatic vessels
MMP: Matrix metalloproteinases
MPO: Myeloperoxidase
MRI: Magnetic resonance imaging
MRP: Macrophage-related protein
mTBI: Mild traumatic brain injury
Myd88: Myeloid differentiation primary response 88
NADPH: Nicotinamide adenine dinucleotide phosphate
NLRP1: NLR Family Pyrin Domain Containing 1
NF-κB: Nuclear factor kappa B
NK: Natural killer
NOX: NADPH oxidase
NT: Neurotrophin
PDPN: Platelet aggregating protein podoplanin
PK: Ligand [11C](R)PK11195
PLX: Pexidartinib
PROX1: Prospero homeobox 1
PRR: Pattern recognition receptors
RAGE: Receptor for advanced glycation end-products
S100B: S100 calcium-binding protein B
sIL: Soluble interleukin receptor
SMC: Smooth muscle cell
TAM: Trauma Associated Microglia
TBI: Traumatic Brain Injury
TCRγδ: T cell receptor gamma/delta
TGF: Transforming growth factor
THINC: Traumatic Head Injury Neuroimaging Classification study (NCT01132937)
TLRs: Toll like receptors
TME: Traumatic Meningeal Enhancement
TMEM: Transmembrane protein
TNF: Tumor necrosis factor
T-reg: Regulatory T cells
UCH-L1: Ubiquitin carboxyl-terminal hydrolase isozyme L1
VEGF-C: Vascular endothelial growth factor C
VEGFR-3: Vascular endothelial growth factor receptor-3
ZO: Zonula Occludens

## References

[1] Injury, G.B.D.T.B. and C. Spinal Cord Injury, Global, regional, and national burden of traumatic brain injury and spinal cord injury, 1990–2016: a systematic analysis for the Global Burden of Disease Study 2016. Lancet Neurol, 2019. 18(1): p. 56–87. 10.1016/S1474-4422(18)30415-0 PMC6291456 30497965

[2] Dewan MC (2018). Estimating the global incidence of traumatic brain injury. J Neurosurg.

[3] Peterson AB, Xu L, Daughtery J, Breiding MJ. Surveillance report of traumatic brain injury-related emergency department visits, hospitalizations, and deaths, United States, 2014. United States, 2019. 10.15585/mmwr.ss6609a1 PMC5829835 28301451

[4] Faul MW, Marlena M, Xu L, Coronado VG, Marlena M, Xu Likang, Coronado VG. Traumatic brain injury in the United States. Atlanta, GA: national Center for injury Prevention and Control, Centers for disease Control and Prevention. 2010.

[5] Gardner RC (2018). Geriatric traumatic brain injury: epidemiology, outcomes, knowledge gaps, and future directions. J Neurotrauma.

[6] Papa L, Mendes ME, Braga CF (2012). Mild traumatic brain injury among the geriatric population. Cur Transl Geriatr Exp Gerontol Rep.

[7] Susman M (2002). Traumatic brain injury in the elderly: increased mortality and worse functional outcome at discharge despite lower injury severity. J Trauma Acute Care Surg.

[8] Green RE (2008). Examining moderators of cognitive recovery trajectories after moderate to severe traumatic brain injury. Arch Phys Med Rehabil.

[9] McIntyre A (2013). Mortality among older adults after a traumatic brain injury: a meta-analysis. Brain Inj.

[10] Thompson HJ, McCormick WC, Kagan SH (2006). Traumatic brain injury in older adults: epidemiology, outcomes, and future implications. J Am Geriatr Soc.

[11] Testa JA (2005). Outcome after traumatic brain injury: effects of aging on recovery. Arch Phys Med Rehabil.

[12] CDC. TBI Data and Statistics 2021; https://www.cdc.gov/traumaticbraininjury/data/index.html.

[13] Saatman KE (2008). Classification of traumatic brain injury for targeted therapies. J Neurotrauma.

[14] Simon DW (2017). The far-reaching scope of neuroinflammation after traumatic brain injury. Nat Rev Neurol.

[15] Graham NS, Sharp DJ (2019). Understanding neurodegeneration after traumatic brain injury: from mechanisms to clinical trials in dementia. J Neurol Neurosurg Psychiatry.

[16] Roh JS, Sohn DH (2018). Damage-associated molecular patterns in inflammatory diseases. Immune Netw.

[17] Rai V, Mathews G, Agrawal DK (2022). Translational and clinical significance of DAMPs, PAMPs, and PRRs in trauma-induced inflammation. Arch Clin Biomed Res.

[18] Rodriguez-Baeza A (2003). Morphological features in human cortical brain microvessels after head injury: a three-dimensional and immunocytochemical study. Anat Rec A Discov Mol Cell Evol Biol.

[19] Hay JR (2015). Blood-brain barrier disruption is an early event that may persist for many years after traumatic brain injury in humans. J Neuropathol Exp Neurol.

[20] Blyth BJ (2009). Validation of serum markers for blood-brain barrier disruption in traumatic brain injury. J Neurotrauma.

[21] Ho KM (2014). Prognostic significance of blood-brain barrier disruption in patients with severe nonpenetrating traumatic brain injury requiring decompressive craniectomy. J Neurosurg.

[22] Saw MM (2014). Differential disruption of blood-brain barrier in severe traumatic brain injury. Neurocrit Care.

[23] Stahel PF (2001). Intrathecal levels of complement-derived soluble membrane attack complex (sC5b-9) correlate with blood-brain barrier dysfunction in patients with traumatic brain injury. J Neurotrauma.

[24] Csuka E (1999). IL-10 levels in cerebrospinal fluid and serum of patients with severe traumatic brain injury: relationship to IL-6, TNF-α, TGF-β1 and blood–brain barrier function. J Neuroimmunol.

[25] Tomkins O (2011). Blood-brain barrier breakdown following traumatic brain injury: a possible role in posttraumatic epilepsy. Cardiovasc Psychiatry Neurol.

[26] Johnson VE (2018). Mechanical disruption of the blood-brain barrier following experimental concussion. Acta Neuropathol.

[27] Blyth BJ (2011). Elevated serum ubiquitin carboxy-terminal hydrolase L1 is associated with abnormal blood-brain barrier function after traumatic brain injury. J Neurotrauma.

[28] Roberts DJ (2013). A prospective evaluation of the temporal matrix metalloproteinase response after severe traumatic brain injury in humans. J Neurotrauma.

[29] Kossmann T (1997). Elevated levels of the complement components C3 and factor B in ventricular cerebrospinal fluid of patients with traumatic brain injury. J Neuroimmunol.

[30] Au AK (2012). Cerebrospinal fluid levels of high-mobility group box 1 and cytochrome C predict outcome after pediatric traumatic brain injury. J Neurotrauma.

[31] Laird MD (2014). High mobility group box protein-1 promotes cerebral edema after traumatic brain injury via activation of toll-like receptor 4. Glia.

[32] Gao TL (2012). Expression of HMGB1 and RAGE in rat and human brains after traumatic brain injury. J Trauma Acute Care Surg.

[33] Adamczak SE (2014). Pyroptotic neuronal cell death mediated by the AIM2 inflammasome. J Cereb Blood Flow Metab.

[34] Adamczak S (2012). Inflammasome proteins in cerebrospinal fluid of brain-injured patients as biomarkers of functional outcome: clinical article. J Neurosurg.

[35] Seshadri A (2017). Phenotyping the immune response to trauma: a multiparametric systems immunology approach. Crit Care Med.

[36] Ryan E (2022). Mild-to-severe traumatic brain injury in children: altered cytokines reflect severity. J Neuroinflamm.

[37] Chaban V (2020). Systemic inflammation persists the first year after mild traumatic brain injury: results from the prospective trondheim mild traumatic brain injury study. J Neurotrauma.

[38] Kossmann T (1997). Interleukin-8 released into the cerebrospinal fluid after brain injury is associated with blood-brain barrier dysfunction and nerve growth factor production. J Cereb Blood Flow Metab.

[39] Shiozaki T (2005). Cerebrospinal fluid concentrations of anti-inflammatory mediators in early-phase severe traumatic brain injury. Shock.

[40] Juengst SB (2014). Exploratory associations with tumor necrosis factor-alpha, disinhibition and suicidal endorsement after traumatic brain injury. Brain Behav Immun.

[41] Hayakata T (2004). Changes in CSF S100B and cytokine concentrations in early-phase severe traumatic brain injury. Shock.

[42] Maier B (2006). Delayed elevation of soluble tumor necrosis factor receptors p75 and p55 in cerebrospinal fluid and plasma after traumatic brain injury. Shock.

[43] Ross SA (1994). The presence of tumour necrosis factor in CSF and plasma after severe head injury. Br J Neurosurg.

[44] Yan EB (2014). Post-traumatic hypoxia is associated with prolonged cerebral cytokine production, higher serum biomarker levels, and poor outcome in patients with severe traumatic brain injury. J Neurotrauma.

[45] Waters RJ (2013). Cytokine gene polymorphisms and outcome after traumatic brain injury. J Neurotrauma.

[46] Helmy A (2011). The cytokine response to human traumatic brain injury: temporal profiles and evidence for cerebral parenchymal production. J Cereb Blood Flow Metab.

[47] Liao Y (2013). Oxidative burst of circulating neutrophils following traumatic brain injury in human. PLoS ONE.

[48] Di Battista AP (2016). Inflammatory cytokine and chemokine profiles are associated with patient outcome and the hyperadrenergic state following acute brain injury. J Neuroinflammation.

[49] Devoto C (2017). Inflammation relates to chronic behavioral and neurological symptoms in military personnel with traumatic brain injuries. Cell Transplant.

[50] Ferreira LCB (2014). Increased levels of interleukin-6, -8 and -10 are associated with fatal outcome following severe traumatic brain injury. Brain Inj.

[51] Buttram SD (2007). Multiplex assessment of cytokine and chemokine levels in cerebrospinal fluid following severe pediatric traumatic brain injury: effects of moderate hypothermia. J Neurotrauma.

[52] Chiaretti A (2005). Interleukin 1beta and interleukin 6 relationship with paediatric head trauma severity and outcome. Childs Nerv Syst.

[53] Hadjigeorgiou GM (2005). IL-1RN and IL-1B gene polymorphisms and cerebral hemorrhagic events after traumatic brain injury. Neurology.

[54] Helmy A (2012). Principal component analysis of the cytokine and chemokine response to human traumatic brain injury. PLoS ONE.

[55] Mellergard P (2011). Differences in cerebral extracellular response of interleukin-1beta, interleukin-6, and interleukin-10 after subarachnoid hemorrhage or severe head trauma in humans. Neurosurgery.

[56] Perez-Barcena J (2011). Lack of correlation among intracerebral cytokines, intracranial pressure, and brain tissue oxygenation in patients with traumatic brain injury and diffuse lesions. Crit Care Med.

[57] Hutchinson PJ (2007). Inflammation in human brain injury: intracerebral concentrations of IL-1alpha, IL-1beta, and their endogenous inhibitor IL-1ra. J Neurotrauma.

[58] Sun Y (2019). Elevated serum levels of inflammation-related cytokines in mild traumatic brain injury are associated with cognitive performance. Front Neurol.

[59] Zhao P (2021). Sex differences in cerebral blood flow and serum inflammatory cytokines and their relationships in mild traumatic brain injury. Front Neurol.

[60] Bell MJ (1997). Interleukin-6 and interleukin-10 in cerebrospinal fluid after severe traumatic brain injury in children. J Neurotrauma.

[61] Kossmann T (1996). Interleukin-6 released in human cerebrospinal fluid following traumatic brain injury may trigger nerve growth factor production in astrocytes. Brain Res.

[62] Kossmann T (1995). Intrathecal and serum interleukin-6 and the acute-phase response in patients with severe traumatic brain injuries. Shock.

[63] Maier B (2001). Differential release of interleukines 6, 8, and 10 in cerebrospinal fluid and plasma after traumatic brain injury. Shock.

[64] Shore PM (2004). Continuous versus intermittent cerebrospinal fluid drainage after severe traumatic brain injury in children: effect on biochemical markers. J Neurotrauma.

[65] Singhal A (2002). Association between cerebrospinal fluid interleukin-6 concentrations and outcome after severe human traumatic brain injury. J Neurotrauma.

[66] Winter CD (2004). Raised parenchymal interleukin-6 levels correlate with improved outcome after traumatic brain injury. Brain.

[67] Vedantam A (2021). Early versus late profiles of inflammatory cytokines after mild traumatic brain injury and their association with neuropsychological outcomes. J Neurotrauma.

[68] Magatti M (2023). Systemic immune response in young and elderly patients after traumatic brain injury. Immun Ageing.

[69] Nwachuku EL (2016). Time course of cerebrospinal fluid inflammatory biomarkers and relationship to 6-month neurologic outcome in adult severe traumatic brain injury. Clin Neurol Neurosurg.

[70] Frugier T (2010). In situ detection of inflammatory mediators in post mortem human brain tissue after traumatic injury. J Neurotrauma.

[71] Gill J (2018). Higher exosomal tau, amyloid-beta 42 and IL-10 are associated with mild TBIs and chronic symptoms in military personnel. Brain Inj.

[72] Kirchhoff C (2008). Cerebrospinal IL-10 concentration is elevated in non-survivors as compared to survivors after severe traumatic brain injury. Eur J Med Res.

[73] Schneider Soares FM (2012). Interleukin-10 is an independent biomarker of severe traumatic brain injury prognosis. NeuroImmunoModulation.

[74] Tylicka M (2020). BDNF and IL-8, but not UCHL-1 and IL-11, are markers of brain injury in children caused by mild head trauma. Brain Sci.

[75] Morganti-Kossmann MC (1999). TGF-beta is elevated in the CSF of patients with severe traumatic brain injuries and parallels blood-brain barrier function. J Neurotrauma.

[76] Li Z (2021). M-CSF, IL-6, and TGF-beta promote generation of a new subset of tissue repair macrophage for traumatic brain injury recovery. Sci Adv.

[77] Semple BD (2010). Role of CCL2 (MCP-1) in traumatic brain injury (TBI): evidence from severe TBI patients and CCL2-/- mice. J Cereb Blood Flow Metab.

[78] Stefini R (2008). Chemokine detection in the cerebral tissue of patients with posttraumatic brain contusions. J Neurosurg.

[79] Whalen MJ (2000). Interleukin-8 is increased in cerebrospinal fluid of children with severe head injury. Crit Care Med.

[80] Yatsiv I (2002). Elevated intracranial IL-18 in humans and mice after traumatic brain injury and evidence of neuroprotective effects of IL-18-binding protein after experimental closed head injury. J Cereb Blood Flow Metab.

[81] Lenzlinger PM (2001). Markers for cell-mediated immune response are elevated in cerebrospinal fluid and serum after severe traumatic brain injury in humans. J Neurotrauma.

[82] Li Z (2015). Expression and clinical significance of non-phagocytic cell oxidase 2 and 4 after human traumatic brain injury. Neurol Sci.

[83] Holmin S (1998). Intracerebral inflammation after human brain contusion. Neurosurgery.

[84] Beschorner R (2002). CD14 expression by activated parenchymal microglia/macrophages and infiltrating monocytes following human traumatic brain injury. Acta Neuropathol.

[85] Engel S (2000). Dynamics of microglial activation after human traumatic brain injury are revealed by delayed expression of macrophage-related proteins MRP8 and MRP14. Acta Neuropathol.

[86] Soares HD (1995). Inflammatory leukocytic recruitment and diffuse neuronal degeneration are separate pathological processes resulting from traumatic brain injury. J Neurosci.

[87] Hicks RR (1997). Alterations in BDNF and NT-3 mRNAs in rat hippocampus after experimental brain trauma. Brain Res Mol Brain Res.

[88] Carlos TM (1997). Expression of endothelial adhesion molecules and recruitment of neutrophils after traumatic brain injury in rats. J Leukoc Biol.

[89] Ebert SE (2019). Molecular imaging of neuroinflammation in patients after mild traumatic brain injury: a longitudinal (123) I-CLINDE single photon emission computed tomography study. Eur J Neurol.

[90] Ramlackhansingh AF (2011). Inflammation after trauma: microglial activation and traumatic brain injury. Ann Neurol.

[91] Johnson VE (2013). Inflammation and white matter degeneration persist for years after a single traumatic brain injury. Brain.

[92] Oehmichen M, Theuerkauf I, Meissner C (1999). Is traumatic axonal injury (AI) associated with an early microglial activation? Application of a double-labeling technique for simultaneous detection of microglia and AI. Acta Neuropathol.

[93] Schwab JM (2002). Persistent accumulation of cyclooxygenase-1-expressing microglial cells and macrophages and transient upregulation by endothelium in human brain injury. J Neurosurg.

[94] Bohnert S (2020). TMEM119 as a specific marker of microglia reaction in traumatic brain injury in postmortem examination. Int J Legal Med.

[95] Zhang D (2014). TLR4 inhibitor resatorvid provides neuroprotection in experimental traumatic brain injury: implication in the treatment of human brain injury. Neurochem Int.

[96] Li W (2013). Enhanced cortical expression of myeloid differentiation primary response protein 88 (Myd88) in patients with traumatic brain injury. J Surg Res.

[97] Roquilly A (2017). Role of IL-12 in overcoming the low responsiveness of NK cells to missing self after traumatic brain injury. Clin Immunol.

[98] Mrakovcic-Sutic I (2010). Early changes in frequency of peripheral blood lymphocyte subpopulations in severe traumatic brain-injured patients. Scand J Immunol.

[99] Kong XD (2014). Alterations of natural killer cells in traumatic brain injury. Neurosci Bull.

[100] Li M (2015). Role of regulatory T cell in clinical outcome of traumatic brain injury. Chin Med J.

[101] Gao W (2018). Adrenomedullin reduces secondary injury and improves outcome in rats with fluid percussion brain injury. World Neurosurg.

[102] Jiang Z (2017). Catechin attenuates traumatic brain injury-induced blood–brain barrier damage and improves longer-term neurological outcomes in rats. Exp Physiol.

[103] Alluri H (2016). Attenuation of blood-brain barrier breakdown and hyperpermeability by calpain inhibition. J Biol Chem.

[104] Main BS (2018). Apolipoprotein E4 impairs spontaneous blood brain barrier repair following traumatic brain injury. Mol Neurodegener.

[105] Shigemori Y. et al. Matrix metalloproteinase-9 is associated with blood-brain barrier opening and brain edema formation after cortical contusion in rats. in Brain Edema XIII. 2006. Springer. 10.1007/3-211-30714-1_29 16671440

[106] Nag S, Venugopalan R, Stewart DJ (2007). Increased caveolin-1 expression precedes decreased expression of occludin and claudin-5 during blood-brain barrier breakdown. Acta Neuropathol.

[107] Wang Z-G (2016). bFGF Protects against blood-brain barrier damage through junction protein regulation via PI3K-Akt-Rac1 pathway following traumatic brain injury. Mol Neurobiol.

[108] Dore-Duffy P (2000). Pericyte migration from the vascular wall in response to traumatic brain injury. Microvasc Res.

[109] Needham EJ (2019). The immunological response to traumatic brain injury. J Neuroimmunol.

[110] Alam A (2020). Cellular infiltration in traumatic brain injury. J Neuroinflamm.

[111] Lawson LJ (1990). Heterogeneity in the distribution and morphology of microglia in the normal adult mouse brain. Neuroscience.

[112] Hayes GM, Woodroofe MN, Cuzner ML (1987). Microglia are the major cell type expressing MHC class II in human white matter. J Neurol Sci.

[113] Paolicelli RC (2022). Microglia states and nomenclature: a field at its crossroads. Neuron.

[114] Blinzinger K, Kreutzberg G (1968). Displacement of synaptic terminals from regenerating motoneurons by microglial cells. Z Zellforsch Mikrosk Anat.

[115] Hickman SE (2013). The microglial sensome revealed by direct RNA sequencing. Nat Neurosci.

[116] Cserép C (2020). Microglia monitor and protect neuronal function through specialized somatic purinergic junctions. Science.

[117] Loane DJ (2014). Progressive neurodegeneration after experimental brain trauma: association with chronic microglial activation. J Neuropathol Exp Neurol.

[118] Davalos D (2005). ATP mediates rapid microglial response to local brain injury in vivo. Nat Neurosci.

[119] Roth TL (2014). Transcranial amelioration of inflammation and cell death after brain injury. Nature.

[120] Wang G (2013). Microglia/macrophage polarization dynamics in white matter after traumatic brain injury. J Cereb Blood Flow Metab.

[121] Turtzo LC (2014). Macrophagic and microglial responses after focal traumatic brain injury in the female rat. J Neuroinflammation.

[122] Shen H (2023). Microglia and astrocytes mediate synapse engulfment in a MER tyrosine kinase-dependent manner after traumatic brain injury. Neural Regen Res.

[123] Krukowski K (2021). Novel microglia-mediated mechanisms underlying synaptic loss and cognitive impairment after traumatic brain injury. Brain Behav Immun.

[124] Ritzel RM (2020). Sustained neuronal and microglial alterations are associated with diverse neurobehavioral dysfunction long after experimental brain injury. Neurobiol Dis.

[125] Cao T (2012). Morphological and genetic activation of microglia after diffuse traumatic brain injury in the rat. Neuroscience.

[126] Robinson S (2017). Microstructural and microglial changes after repetitive mild traumatic brain injury in mice. J Neurosci Res.

[127] Witcher KG (2021). Traumatic brain injury causes chronic cortical inflammation and neuronal dysfunction mediated by microglia. J Neurosci.

[128] Izzy S (2019). Time-dependent changes in microglia transcriptional networks following traumatic brain injury. Front Cell Neurosci.

[129] Jin X (2012). Temporal changes in cell marker expression and cellular infiltration in a controlled cortical impact model in adult male C57BL/6 mice. PLoS ONE.

[130] Nimmerjahn A, Kirchhoff F, Helmchen F (2005). Resting microglial cells are highly dynamic surveillants of brain parenchyma in vivo. Science.

[131] Zhang Z (2012). Immunolocalization of Toll-like receptors 2 and 4 as well as their endogenous ligand, heat shock protein 70, in rat traumatic brain injury. NeuroImmunoModulation.

[132] Chen G (2008). Progesterone administration modulates TLRs/NF-kappaB signaling pathway in rat brain after cortical contusion. Ann Clin Lab Sci.

[133] Hang CH (2005). Concomitant upregulation of nuclear factor-kB activity, proinflammatory cytokines and ICAM-1 in the injured brain after cortical contusion trauma in a rat model. Neurol India.

[134] Yu ZQ, Zha JH (2012). Genetic ablation of toll-like receptor 2 reduces secondary brain injury caused by cortical contusion in mice. Ann Clin Lab Sci.

[135] Ahmad A (2013). Absence of TLR4 reduces neurovascular unit and secondary inflammatory process after traumatic brain injury in mice. PLoS ONE.

[136] Fourgeaud L (2016). TAM receptors regulate multiple features of microglial physiology. Nature.

[137] Kumar A (2016). Microglial/macrophage polarization dynamics following traumatic brain injury. J Neurotrauma.

[138] Dohi K (2010). Gp91phox (NOX2) in classically activated microglia exacerbates traumatic brain injury. J Neuroinflamm.

[139] Henry RJ (2020). Microglial depletion with CSF1R inhibitor during chronic phase of experimental traumatic brain injury reduces neurodegeneration and neurological deficits. J Neurosci.

[140] Escartin C (2021). Reactive astrocyte nomenclature, definitions, and future directions. Nat Neurosci.

[141] Allen NJ (2012). Astrocyte glypicans 4 and 6 promote formation of excitatory synapses via GluA1 AMPA receptors. Nature.

[142] Tsai HH (2012). Regional astrocyte allocation regulates CNS synaptogenesis and repair. Science.

[143] Chung WS (2013). Astrocytes mediate synapse elimination through MEGF10 and MERTK pathways. Nature.

[144] Molofsky AV (2014). Astrocyte-encoded positional cues maintain sensorimotor circuit integrity. Nature.

[145] Alvarez JI (2011). The Hedgehog pathway promotes blood-brain barrier integrity and CNS immune quiescence. Science.

[146] Faulkner JR (2004). Reactive astrocytes protect tissue and preserve function after spinal cord injury. J Neurosci.

[147] Myer DJ (2006). Essential protective roles of reactive astrocytes in traumatic brain injury. Brain.

[148] Bush TG (1999). Leukocyte infiltration, neuronal degeneration, and neurite outgrowth after ablation of scar-forming, reactive astrocytes in adult transgenic mice. Neuron.

[149] Villapol S, Byrnes KR, Symes AJ (2014). Temporal dynamics of cerebral blood flow, cortical damage, apoptosis, astrocyte-vasculature interaction and astrogliosis in the pericontusional region after traumatic brain injury. Front Neurol.

[150] Glober NK (2019). Acetazolamide treatment prevents redistribution of astrocyte aquaporin 4 after murine traumatic brain injury. Neurosci J.

[151] Jayakumar AR (2014). Activation of NF-kappaB mediates astrocyte swelling and brain edema in traumatic brain injury. J Neurotrauma.

[152] Gorse KM (2018). Transient receptor potential melastatin 4 induces astrocyte swelling but not death after diffuse traumatic brain injury. J Neurotrauma.

[153] Gupta RK, Prasad S (2013). Early down regulation of the glial Kir4.1 and GLT-1 expression in pericontusional cortex of the old male mice subjected to traumatic brain injury. Biogerontology.

[154] Gupta RK, Prasad S (2016). Age-dependent alterations in the interactions of NF-kappaB and N-myc with GLT-1/EAAT2 promoter in the pericontusional cortex of mice subjected to traumatic brain injury. Mol Neurobiol.

[155] Rao VL (1998). Traumatic brain injury down-regulates glial glutamate transporter (GLT-1 and GLAST) proteins in rat brain. J Neurochem.

[156] Li YH (2015). Effects of mild induced hypothermia on hippocampal connexin 43 and glutamate transporter 1 expression following traumatic brain injury in rats. Mol Med Rep.

[157] Jiang H (2018). Toll-Like receptor 4 knockdown attenuates brain damage and neuroinflammation after traumatic brain injury via inhibiting neuronal autophagy and astrocyte activation. Cell Mol Neurobiol.

[158] Herx LM, Yong VW (2001). Interleukin-1β is required for the early evolution of reactive astrogliosis following CNS lesion. J Neuropathol Exp Neurol.

[159] Wang XY (2015). Endogenous TGFbeta1 plays a crucial role in functional recovery after traumatic brain injury associated with smad3 signal in rats. Neurochem Res.

[160] Toutonji A (2021). Chronic complement dysregulation drives neuroinflammation after traumatic brain injury: a transcriptomic study. Acta Neuropathol Commun.

[161] Arneson D (2018). Single cell molecular alterations reveal target cells and pathways of concussive brain injury. Nat Commun.

[162] Todd BP (2021). Traumatic brain injury results in unique microglial and astrocyte transcriptomes enriched for type I interferon response. J Neuroinflamm.

[163] Arneson D (2022). Systems spatiotemporal dynamics of traumatic brain injury at single-cell resolution reveals humanin as a therapeutic target. Cell Mol Life Sci.

[164] Xing J (2022). Single-cell RNA sequencing reveals cellular and transcriptional changes associated with traumatic brain injury. Front Genet.

[165] Liddelow SA (2017). Neurotoxic reactive astrocytes are induced by activated microglia. Nature.

[166] Clarke LE (2018). Normal aging induces A1-like astrocyte reactivity. Proc Natl Acad Sci USA.

[167] Guttenplan KA (2021). Neurotoxic reactive astrocytes induce cell death via saturated lipids. Nature.

[168] Prescott K (2023). Blocking formation of neurotoxic reactive astrocytes is beneficial following stroke. biorxiv.

[169] Morganti-Kossmann MC (2019). The complexity of neuroinflammation consequent to traumatic brain injury: from research evidence to potential treatments. Acta Neuropathol.

[170] Simon DW (2017). The far-reaching scope of neuroinflammation after traumatic brain injury. Nat Rev Neurol.

[171] Weller RO (2018). The meninges as barriers and facilitators for the movement of fluid, cells and pathogens related to the rodent and human CNS. Acta Neuropathol.

[172] Nabeshima S (1975). Junctions in the meninges and marginal glia. J Comp Neurol.

[173] Louveau A (2015). Structural and functional features of central nervous system lymphatic vessels. Nature.

[174] Absinta M (2017). Human and nonhuman primate meninges harbor lymphatic vessels that can be visualized noninvasively by MRI. Elife.

[175] Aspelund A (2015). A dural lymphatic vascular system that drains brain interstitial fluid and macromolecules. J Exp Med.

[176] Visanji NP, Lang AE, Munoz DG (2018). Lymphatic vasculature in human dural superior sagittal sinus: Implications for neurodegenerative proteinopathies. Neurosci Lett.

[177] Alcolado R (1988). The cranial arachnoid and pia mater in man: anatomical and ultrastructural observations. Neuropathol Appl Neurobiol.

[178] Vandenabeele F, Creemers J, Lambrichts I (1996). Ultrastructure of the human spinal arachnoid mater and dura mater. J Anat.

[179] Balin BJ (1986). Avenues for entry of peripherally administered protein to the central nervous system in mouse, rat, and squirrel monkey. J Comp Neurol.

[180] Vinas FC (1996). Microsurgical anatomy of the arachnoidal trabecular membranes and cisterns at the level of the tentorium. Neurol Res.

[181] Dorrier CE (2022). Emerging roles for CNS fibroblasts in health, injury and disease. Nat Rev Neurosci.

[182] Spector R, Robert Snodgrass S, Johanson CE (2015). A balanced view of the cerebrospinal fluid composition and functions: focus on adult humans. Exp Neurol.

[183] Hannocks MJ (2018). Molecular characterization of perivascular drainage pathways in the murine brain. J Cereb Blood Flow Metab.

[184] Pietila R (2023). Molecular anatomy of adult mouse leptomeninges. Neuron.

[185] Vanlandewijck M (2018). A molecular atlas of cell types and zonation in the brain vasculature. Nature.

[186] Van Hove H (2019). A single-cell atlas of mouse brain macrophages reveals unique transcriptional identities shaped by ontogeny and tissue environment. Nat Neurosci.

[187] Brochner CB, Holst CB, Mollgard K (2015). Outer brain barriers in rat and human development. Front Neurosci.

[188] Natale G, Bocci G, Ribatti D (2017). Scholars and scientists in the history of the lymphatic system. J Anat.

[189] Mascagni P. Vasorum lymphaticorum corporis humani historia et ichnographia. (No Title), 1787.

[190] Sandrone S (2019). A (delayed) history of the brain lymphatic system. Nat Med.

[191] Izen RM (2018). Postnatal development of lymphatic vasculature in the brain meninges. Dev Dyn.

[192] Ahn JH (2019). Meningeal lymphatic vessels at the skull base drain cerebrospinal fluid. Nature.

[193] Jacob L (2019). Anatomy and function of the vertebral column lymphatic network in mice. Nat Commun.

[194] Jacob L (2022). Conserved meningeal lymphatic drainage circuits in mice and humans. J Exp Med.

[195] Wigle JT, Oliver G (1999). Prox1 function is required for the development of the murine lymphatic system. Cell.

[196] Oliver G, Srinivasan RS (2010). Endothelial cell plasticity: how to become and remain a lymphatic endothelial cell. Development.

[197] Escobedo N, Oliver G (2016). Lymphangiogenesis: origin, specification, and cell fate determination. Annu Rev Cell Dev Biol.

[198] Francois M (2008). Sox18 induces development of the lymphatic vasculature in mice. Nature.

[199] Wigle JT (2002). An essential role for Prox1 in the induction of the lymphatic endothelial cell phenotype. EMBO J.

[200] You LR (2005). Suppression of Notch signalling by the COUP-TFII transcription factor regulates vein identity. Nature.

[201] Lin FJ (2010). Direct transcriptional regulation of neuropilin-2 by COUP-TFII modulates multiple steps in murine lymphatic vessel development. J Clin Invest.

[202] Srinivasan RS (2010). The nuclear hormone receptor Coup-TFII is required for the initiation and early maintenance of Prox1 expression in lymphatic endothelial cells. Genes Dev.

[203] Oliver G (2004). Lymphatic vasculature development. Nat Rev Immunol.

[204] Tammela T, Alitalo K (2010). Lymphangiogenesis: molecular mechanisms and future promise. Cell.

[205] Makinen T (2001). Inhibition of lymphangiogenesis with resulting lymphedema in transgenic mice expressing soluble VEGF receptor-3. Nat Med.

[206] Karpanen T (2006). Lymphangiogenic growth factor responsiveness is modulated by postnatal lymphatic vessel maturation. Am J Pathol.

[207] Karkkainen MJ (2004). Vascular endothelial growth factor C is required for sprouting of the first lymphatic vessels from embryonic veins. Nat Immunol.

[208] Yoshimatsu Y, Miyazaki H, Watabe T (2016). Roles of signaling and transcriptional networks in pathological lymphangiogenesis. Adv Drug Deliv Rev.

[209] Karpanen T, Alitalo K (2008). Molecular biology and pathology of lymphangiogenesis. Annu Rev Pathol.

[210] Antila S (2017). Development and plasticity of meningeal lymphatic vessels. J Exp Med.

[211] Rustenhoven J (2021). Functional characterization of the dural sinuses as a neuroimmune interface. Cell.

[212] Balint L (2019). Lymph flow induces the postnatal formation of mature and functional meningeal lymphatic vessels. Front Immunol.

[213] Oliver G (2020). The lymphatic vasculature in the 21(st) century: novel functional roles in homeostasis and disease. Cell.

[214] Hammerling B (2006). The complexus adhaerens of mammalian lymphatic endothelia revisited: a junction even more complex than hitherto thought. Cell Tissue Res.

[215] Yao LC (2012). Plasticity of button-like junctions in the endothelium of airway lymphatics in development and inflammation. Am J Pathol.

[216] Jannaway M (2023). VEGFR3 is required for button junction formation in lymphatic vessels. Cell Rep.

[217] Baluk P (2007). Functionally specialized junctions between endothelial cells of lymphatic vessels. J Exp Med.

[218] Pflicke H, Sixt M (2009). Preformed portals facilitate dendritic cell entry into afferent lymphatic vessels. J Exp Med.

[219] Johnson LA (2017). Dendritic cells enter lymph vessels by hyaluronan-mediated docking to the endothelial receptor LYVE-1. Nat Immunol.

[220] Baluk P, McDonald DM (2022). Buttons and zippers: endothelial junctions in lymphatic vessels. Cold Spring Harb Perspect Med.

[221] Weigel C, Bellaci J, Spiegel S (2023). Sphingosine-1-phosphate and its receptors in vascular endothelial and lymphatic barrier function. J Biol Chem.

[222] Petrova TV, Koh GY (2020). Biological functions of lymphatic vessels. Science.

[223] Louveau A (2017). Understanding the functions and relationships of the glymphatic system and meningeal lymphatics. J Clin Invest.

[224] Kuo PH (2018). Meningeal lymphatic vessel flow runs countercurrent to venous flow in the superior sagittal sinus of the human brain. Tomography.

[225] Louveau A (2018). CNS lymphatic drainage and neuroinflammation are regulated by meningeal lymphatic vasculature. Nat Neurosci.

[226] Albayram MS (2022). Non-invasive MR imaging of human brain lymphatic networks with connections to cervical lymph nodes. Nat Commun.

[227] Asano K (2020). Pre-collecting lymphatic vessels form detours following obstruction of lymphatic flow and function as collecting lymphatic vessels. PLoS ONE.

[228] Shah T (2023). Arachnoid granulations are lymphatic conduits that communicate with bone marrow and dura-arachnoid stroma. J Exp Med.

[229] Smyth, L.C.D., et al., *Identification of direct connections between the dura and the brain.* Nature, 2024. 10.1038/s41586-023-06993-7 PMC11254388 38326613

[230] Utz SG (2020). Early fate defines microglia and non-parenchymal brain macrophage development. Cell.

[231] Alves de Lima K (2020). Meningeal γδ T cells regulate anxiety-like behavior via IL-17a signaling in neurons. Nat Immunol.

[232] Mazzitelli JA (2022). Cerebrospinal fluid regulates skull bone marrow niches via direct access through dural channels. Nat Neurosci.

[233] Cugurra A (2021). Skull and vertebral bone marrow are myeloid cell reservoirs for the meninges and CNS parenchyma. Science.

[234] Brioschi S (2021). Heterogeneity of meningeal B cells reveals a lymphopoietic niche at the CNS borders. Science.

[235] Herisson F (2018). Direct vascular channels connect skull bone marrow and the brain surface enabling myeloid cell migration. Nat Neurosci.

[236] Mrdjen D (2018). High-dimensional single-cell mapping of central nervous system immune cells reveals distinct myeloid subsets in health, aging, and disease. Immunity.

[237] Ajami B (2018). Single-cell mass cytometry reveals distinct populations of brain myeloid cells in mouse neuroinflammation and neurodegeneration models. Nat Neurosci.

[238] Drieu A (2022). Parenchymal border macrophages regulate the flow dynamics of the cerebrospinal fluid. Nature.

[239] Da Mesquita S (2021). Aging-associated deficit in CCR7 is linked to worsened glymphatic function, cognition, neuroinflammation, and beta-amyloid pathology. Sci Adv.

[240] Da Mesquita S (2021). Meningeal lymphatics affect microglia responses and anti-Abeta immunotherapy. Nature.

[241] Gadani SP (2017). Characterization of meningeal type 2 innate lymphocytes and their response to CNS injury. J Exp Med.

[242] Gate D (2020). Clonally expanded CD8 T cells patrol the cerebrospinal fluid in Alzheimer's disease. Nature.

[243] Schafflick D (2020). Integrated single cell analysis of blood and cerebrospinal fluid leukocytes in multiple sclerosis. Nat Commun.

[244] Schlager C (2016). Effector T-cell trafficking between the leptomeninges and the cerebrospinal fluid. Nature.

[245] Merlini A (2022). Distinct roles of the meningeal layers in CNS autoimmunity. Nat Neurosci.

[246] Lapenna A, De Palma M, Lewis CE (2018). Perivascular macrophages in health and disease. Nat Rev Immunol.

[247] Chakarov S (2019). Two distinct interstitial macrophage populations coexist across tissues in specific subtissular niches. Science.

[248] Wang AZ (2022). Single-cell profiling of human dura and meningioma reveals cellular meningeal landscape and insights into meningioma immune response. Genome Med.

[249] Masuda T (2022). Specification of CNS macrophage subsets occurs postnatally in defined niches. Nature.

[250] Rojo R (2019). Deletion of a Csf1r enhancer selectively impacts CSF1R expression and development of tissue macrophage populations. Nat Commun.

[251] Munro DAD (2020). CNS macrophages differentially rely on an intronic Csf1r enhancer for their development. Development.

[252] Rua R (2019). Infection drives meningeal engraftment by inflammatory monocytes that impairs CNS immunity. Nat Immunol.

[253] Rebejac J (2022). Meningeal macrophages protect against viral neuroinfection. Immunity.

[254] Mundt S (2019). Conventional DCs sample and present myelin antigens in the healthy CNS and allow parenchymal T cell entry to initiate neuroinflammation. Sci Immunol.

[255] Anandasabapathy N (2011). Flt3L controls the development of radiosensitive dendritic cells in the meninges and choroid plexus of the steady-state mouse brain. J Exp Med.

[256] Ugur M (2023). Lymph node medulla regulates the spatiotemporal unfolding of resident dendritic cell networks. Immunity.

[257] Clarkson BD (2015). CCR2-dependent dendritic cell accumulation in the central nervous system during early effector experimental autoimmune encephalomyelitis is essential for effector T cell restimulation in situ and disease progression. J Immunol.

[258] Hatterer E (2006). How to drain without lymphatics? Dendritic cells migrate from the cerebrospinal fluid to the B-cell follicles of cervical lymph nodes. Blood.

[259] Hatterer E (2008). Cerebrospinal fluid dendritic cells infiltrate the brain parenchyma and target the cervical lymph nodes under neuroinflammatory conditions. PLoS ONE.

[260] Carson MJ (1999). Disproportionate recruitment of CD8+ T cells into the central nervous system by professional antigen-presenting cells. Am J Pathol.

[261] Hu X (2020). Meningeal lymphatic vessels regulate brain tumor drainage and immunity. Cell Res.

[262] Hsu M (2022). Neuroinflammation creates an immune regulatory niche at the meningeal lymphatic vasculature near the cribriform plate. Nat Immunol.

[263] Goldmann J (2006). T cells traffic from brain to cervical lymph nodes via the cribroid plate and the nasal mucosa. J Leukoc Biol.

[264] Bromley SK, Thomas SY, Luster AD (2005). Chemokine receptor CCR7 guides T cell exit from peripheral tissues and entry into afferent lymphatics. Nat Immunol.

[265] Debes GF (2005). Chemokine receptor CCR7 required for T lymphocyte exit from peripheral tissues. Nat Immunol.

[266] Kivisakk P (2003). Human cerebrospinal fluid central memory CD4+ T cells: evidence for trafficking through choroid plexus and meninges via P-selectin. Proc Natl Acad Sci USA.

[267] de Graaf MT (2011). Central memory CD4+ T cells dominate the normal cerebrospinal fluid. Cytometry B Clin Cytom.

[268] Filiano AJ (2016). Unexpected role of interferon-gamma in regulating neuronal connectivity and social behaviour. Nature.

[269] Derecki NC (2010). Regulation of learning and memory by meningeal immunity: a key role for IL-4. J Exp Med.

[270] Pasciuto E (2020). Microglia require CD4 T cells to complete the fetal-to-adult transition. Cell.

[271] Ziv Y (2006). Immune cells contribute to the maintenance of neurogenesis and spatial learning abilities in adulthood. Nat Neurosci.

[272] Herz J (2021). GABAergic neuronal IL-4R mediates T cell effect on memory. Neuron.

[273] Sankowski R (2019). Endogenous retroviruses are associated with hippocampus-based memory impairment. Proc Natl Acad Sci USA.

[274] Kipnis J (2004). T cell deficiency leads to cognitive dysfunction: implications for therapeutic vaccination for schizophrenia and other psychiatric conditions. Proc Natl Acad Sci.

[275] Radjavi A, Smirnov I, Kipnis J (2014). Brain antigen-reactive CD4+ T cells are sufficient to support learning behavior in mice with limited T cell repertoire. Brain Behav Immun.

[276] Jain RW, Yong VW (2022). B cells in central nervous system disease: diversity, locations and pathophysiology. Nat Rev Immunol.

[277] Howell OW (2011). Meningeal inflammation is widespread and linked to cortical pathology in multiple sclerosis. Brain.

[278] Machado-Santos J (2018). The compartmentalized inflammatory response in the multiple sclerosis brain is composed of tissue-resident CD8+ T lymphocytes and B cells. Brain.

[279] Schafflick D (2021). Single-cell profiling of CNS border compartment leukocytes reveals that B cells and their progenitors reside in non-diseased meninges. Nat Neurosci.

[280] Korin B (2017). High-dimensional, single-cell characterization of the brain's immune compartment. Nat Neurosci.

[281] Wang Y (2021). Early developing B cells undergo negative selection by central nervous system-specific antigens in the meninges. Immunity.

[282] Fitzpatrick Z (2020). Gut-educated IgA plasma cells defend the meningeal venous sinuses. Nature.

[283] Lee AL (2020). Advanced imaging of traumatic brain injury. Korean J Neurotrauma.

[284] Coles JP (2007). Imaging after brain injury. Br J Anaesth.

[285] Yuh EL (2021). Pathological computed tomography features associated with adverse outcomes after mild traumatic brain injury: a TRACK-TBI study with external validation in CENTER-TBI. JAMA Neurol.

[286] Bischof GN, Cross DJ (2023). Brain trauma imaging. J Nucl Med.

[287] Lee EK (2016). Importance of contrast-enhanced fluid-attenuated inversion recovery magnetic resonance imaging in various intracranial pathologic conditions. Korean J Radiol.

[288] Bahsoun MA (2022). FLAIR MRI biomarkers of the normal appearing brain matter are related to cognition. Neuroimage Clin.

[289] Turtzo LC (2020). Meningeal blood-brain barrier disruption in acute traumatic brain injury. Brain Commun.

[290] Kenney K (2016). Cerebral vascular injury in traumatic brain injury. Exp Neurol.

[291] Kim SC (2014). Contrast-enhanced FLAIR (fluid-attenuated inversion recovery) for evaluating mild traumatic brain injury. PLoS ONE.

[292] Chiara Ricciardi M (2017). Trauma-specific brain abnormalities in suspected mild traumatic brain injury patients identified in the first 48 hours after injury: a blinded magnetic resonance imaging comparative study including suspected acute minor stroke patients. J Neurotrauma.

[293] Russo MV, Latour LL, McGavern DB (2018). Distinct myeloid cell subsets promote meningeal remodeling and vascular repair after mild traumatic brain injury. Nat Immunol.

[294] Livingston WS (2017). Differential gene expression associated with meningeal injury in acute mild traumatic brain injury. J Neurotrauma.

[295] Davis TS (2020). ––-Comparison of T1-Post and FLAIR-Post MRI for identification of traumatic meningeal enhancement in traumatic brain injury patients. PLoS ONE.

[296] Rizk T (2020). Traumatic microbleeds persist for up to five years following traumatic brain injury despite resolution of other acute findings on MRI. Brain Inj.

[297] Koh BI (2020). VEGFR2 signaling drives meningeal vascular regeneration upon head injury. Nat Commun.

[298] Baban B (2021). AMPK induces regulatory innate lymphoid cells after traumatic brain injury. JCI Insight.

[299] Ineichen BV (2022). Leptomeningeal enhancement in multiple sclerosis and other neurological diseases: a systematic review and meta-analysis. Neuroimage Clin.

[300] Absinta M (2017). Leptomeningeal gadolinium enhancement across the spectrum of chronic neuroinflammatory diseases. Neurology.

[301] Qureshi AI (2015). Prevalence of and factors associated with dural thickness in patients with mild cognitive impairment and Alzheimer's Disease. J Vasc Interv Neurol.

[302] Bolte AC (2023). The meningeal transcriptional response to traumatic brain injury and aging. Elife.

[303] Liu M (2023). Exogenous interleukin 33 enhances the brain's lymphatic drainage and toxic protein clearance in acute traumatic brain injury mice. Acta Neuropathol Commun.

[304] Liao J (2023). Improving the function of meningeal lymphatic vessels to promote brain edema absorption after traumatic brain injury. J Neurotrauma.

[305] McNamara EH (2022). Meningeal and visual pathway magnetic resonance imaging analysis after single and repetitive closed-head impact model of engineered rotational acceleration (CHIMERA)-induced disruption in male and female mice. J Neurotrauma.

[306] Lin J (2023). Meningeal lymphatics restoration and neurovascular protection synergistically improve traumatic brain injury treatment. Chem Eng J.

[307] Shimada R, Tatara Y, Kibayashi K (2022). Gene expression in meningeal lymphatic endothelial cells following traumatic brain injury in mice. PLoS ONE.

[308] Tong S (2023). Nano-plumber reshapes glymphatic-lymphatic system to sustain microenvironment homeostasis and improve long-term prognosis after traumatic brain injury. Adv Sci.

[309] Bolte AC (2020). Meningeal lymphatic dysfunction exacerbates traumatic brain injury pathogenesis. Nat Commun.

[310] Chen J (2020). Meningeal lymphatics clear erythrocytes that arise from subarachnoid hemorrhage. Nat Commun.

[311] Daglas M (2019). Activated CD8(+) T cells cause long-term neurological impairment after traumatic brain injury in mice. Cell Rep.

[312] Wojciechowski S (2020). Developmental dysfunction of the central nervous system lymphatics modulates the adaptive neuro-immune response in the perilesional cortex in a mouse model of traumatic brain injury. Front Immunol.

[313] Vokali E (2020). Lymphatic endothelial cells prime naive CD8(+) T cells into memory cells under steady-state conditions. Nat Commun.

[314] Tsymbalyuk O (2022). Traumatic brain injury alters dendritic cell differentiation and distribution in lymphoid and non-lymphoid organs. J Neuroinflamm.

[315] Israelsson C (2014). Interacting chemokine signals regulate dendritic cells in acute brain injury. PLoS ONE.

[316] Moro F (2022). Ageing is associated with maladaptive immune response and worse outcome after traumatic brain injury. Brain Commun.

[317] Buenaventura RG (2023). Traumatic brain injury induces an adaptive immune response in the meningeal transcriptome that is amplified by aging. Front Neurosci.

[318] Morganti JM (2015). CCR2 antagonism alters brain macrophage polarization and ameliorates cognitive dysfunction induced by traumatic brain injury. J Neurosci.

[319] Somebang K (2021). CCR2 deficiency alters activation of microglia subsets in traumatic brain injury. Cell Rep.

[320] Hsieh CL (2014). CCR2 deficiency impairs macrophage infiltration and improves cognitive function after traumatic brain injury. J Neurotrauma.

[321] Mastorakos P (2021). Antimicrobial immunity impedes CNS vascular repair following brain injury. Nat Immunol.

